# Insights From Developmental and Formative Evaluations: Developing and Implementing a Limited-Term Housing Mobility Program

**DOI:** 10.1177/0193841X261468394

**Published:** 2026-07-16

**Authors:** Jee Young Lee, Jason Reece, Rachel Garshick Kleit

**Affiliations:** 1 City & Regional Planning, Knowlton School, College of Engineering, 2647 The Ohio State University, Columbus, OH, USA

**Keywords:** housing mobility program, formative evaluation, developmental evaluation, affordable housing, program implementation

## Abstract

This paper aims to demonstrate how evaluation methods can inform the development of a program, using the example of a novel housing mobility program. The limited availability of Housing Choice Vouchers (HCVs) and other affordable housing assistance restricts the ability to address US national housing needs; millions more households qualify for HCVs than receive them. Those with HCVs encounter challenges in using vouchers in so-called high-opportunity areas. To address the dearth of HCVs and difficulty using them, a collaboration between faculty at Ohio State University and the community developed a new housing mobility program to increase the affordable housing supply, engaging stakeholders to understand the needs of low-income renters who are less likely to obtain an HCV. This paper outlines the Families Flourish program’s development using both developmental and formative evaluations, examining the progress of the implementation and immediate outcomes using mixed methods. The developmental evaluation process required collaboration with a wide variety of stakeholders, partners, and the project team. According to the formative evaluation of the pilot program, participants’ experiences suggested that enhanced housing security was associated with improvements in physical and mental health for parents and their children, and that a more stable and safe neighborhood environment was similarly associated with gains in employment, education, and income. Throughout the process of evaluation, particularly the developmental evaluation, the evaluator’s communication and collaboration skills were essential to the methodology. The study concludes with lessons learned, as well as recommendations for policy and directions for future research.

## Introduction

Housing mobility programs aim to diminish inequalities and encourage fairer access to improved living conditions by expanding housing choice for low-income communities and communities of color (Chetty et al., 2016; DeLuca et al., 2016). However, the scarcity of federal Housing Choice Vouchers (HCVs) and other forms of housing assistance limits the capacity of such programs to meet housing needs within the US (Moore, 2016). According to the GAP report from the National Low Income Housing Coalition (2024), approximately 17.9 million renter households were classified as very low-income or extremely low-income in 2022, making them technically eligible for HCVs (households earning at or below 50% of the area median income). However, only about 5.2 million households receive federal rental assistance, including 2.3 million who benefit from HCVs (Center on Budget and Policy Priorities, 2022; Congressional Research Service, 2023).

Voucher programs often have waitlists that extend over several years (Ellen, 2020). In the region of concern in this paper, Central Ohio, the local newspaper reported that the region’s public housing authority had 26,000 applications on the waiting list in 2023 but had resources to fund only approximately 13,600 vouchers (Gill & Ferenchik, 2023). In the local county, 34,000 people are currently waiting for a voucher (King, 2024). Nationally, HCV holders also encounter difficulties utilizing vouchers in high-opportunity areas (McClure et al., 2015) due to cost constraints and landlord resistance to taking HCVs. Housing mobility programs have been designed to help HCV holders move to such areas (Center on Budget and Policy Priorities, 2019); however, a drawback of these programs is their dependency on traditional federal housing assistance programs to subsidize participants’ long-term housing. When a family’s income increases, so does their rent, which can discourage income growth and hinder economic mobility.

Despite evidence of the positive impacts of housing mobility programs on children’s development and life outcomes (Chetty et al., 2016; Chyn et al., 2025), as well as physical and mental health (Krieger & Higgins, 2002; Martin et al., 2019; Sandel et al., 2004; Singh et al., 2019; Stahre et al., 2015), they are consistently criticized. For example, the concept of the “geography of opportunity” has been criticized as reflecting norms of Whiteness at the neighborhood scale (Goetz et al., 2020). Additionally, criticism of many housing mobility programs cite evidence of discrimination facing youth of color (particularly for young boys of color) in predominately White schools and neighborhoods (Rudolph et al., 2021). Scholars have also criticized mobility programs for not addressing structural drivers of segregation, for imposing the burden of integration of households of color and potentially hastening gentrification in urban areas (Goetz, 2018).

To address this criticism, a different type of housing mobility program was designed in collaboration with the community, involving an initial process that thoroughly engaged stakeholders to understand the desires of the target communities. Thus, as an alternative to traditional housing mobility programs, faculty at Ohio State University and community partners collaborated to create the Families Flourish Program^1^ that generates opportunities for residential and financial stability using a limited-term rental subsidy model rather than HCVs whose term are indefinite. The goals were to increase the supply of affordable housing, improve the housing stability of participating families, and improve life outcomes for children and their families.

While current housing mobility literature primarily focuses on individuals with HCVs and traditional public housing, this Program targets the approximately 6.9 million very low-income renter households (those earning between 30% and 50% of the area median income) who qualify for but are unlikely to obtain an HCV, since 75% of HCVs are allocated to extremely low-income households (those earning under 30% of the area median income, as mandated by law) (U.S. Department of Housing and Urban Development, n.d). We focus on this larger yet often overlooked population, which faces housing problems such as instability and lower quality housing.

Since the 1970s, spanning several decades, various housing mobility programs have emerged, including the Gautreaux Program (1976–1998), the Moving to Opportunity (MTO) demonstration (1994–2012), the Creating Moves to Opportunity (2018–2020), and Community Choice Demonstration (2022–2028). The Gautreaux Program assisted approximately 7,000 families in moving to low-poverty, majority White suburban neighborhoods over twenty years (Rosenbaum et al., 2002), producing positive outcomes for families, including improved adult employment and youth educational outcomes (Rosenbaum, 1991, 1995). The critique of Gautreaux was that it was not an experimental design, so it was difficult to ascribe outcomes to the program. MTO, in contrast, was designed as a decade-long social science experiment comparing outcomes among three groups: households that received HCVs and moved to low-poverty areas (experimental group), households that received HCVs and moved to neighborhoods of their choosing, and households that did not receive HCVs (Sanbonmatsu et al., 2011). Initial evaluations of MTO demonstrated strong mental health benefits and positive outcomes for some children, although within the span of the program’s implementation, there were not significant economic impacts on households (Sanbonmatsu et al., 2011). The impacts of MTO were reappraised after 20 years in longitudinal analysis of adult outcomes for children in the program, revealing significant positive impacts on economic well-being, educational attainment and health for adults who were children in the MTO program (Chetty et al., 2016).

More recent housing mobility programs have emphasized the role of housing mobility and mobility counseling (assisting families in relocation) to improve family outcomes (DeLuca et al., 2023). Creating Moves to Opportunity, which helps families move to high-opportunity areas, provides search assistance, landlord outreach, and short-term financial supports. The study found that families who received these services were more likely to move to high-opportunity areas than families who did not receive them (Bergman et al., 2023).

The most recent large-scale initiative, the Community Choice Demonstration, evaluates the effectiveness of mobility-related services in helping families with HCVs move to opportunity areas. Its comprehensive services include housing search assistance, financial assistance, and both pre- and post-move services (Lubell et al., 2024). Pre-move services address potential barriers and discuss opportunity areas, while post-move services include a one-month check-in and annual follow-ups (Lubell et al., 2024). In a period of escalating housing needs across the nation, innovative solutions are needed to provide stable affordable housing in high-opportunity areas.

To develop an innovative program that adapts the framework of existing housing mobility programs to this target population, the authors worked with community representatives to conduct both developmental and formative evaluations. This paper aims to demonstrate how evaluation methods can inform the development of a program, using the example of a novel housing mobility program. Although traditional housing mobility programs offer established principles and practices for programs where subsidies are long-term, the rental assistance for the Program is only for three years. Thus, the short-term nature of the Program means that adjustments were necessary.

This study contributes in two ways: methodologically and policy-wise. First, it demonstrates how to integrate evaluation into program design and offers insights for practitioners interested in developing new programs using developmental and formative evaluations. The study elucidates how the program responded to the formative evaluation in real time as well as to unexpected challenges using information from both participants and service providers. Second, it explains the motivation for this new type of housing mobility program, what it entails, and how it addresses the current gaps in housing policy.

The remainder of this paper is organized as follows. First, we describe the methods, including data collection and analysis. Second, we outline the program’s rationale and foundations, including the timeline and program team. Third, we present the program design and development phase. In this section, we highlight the developmental evaluation, focusing on how we adapted principles of housing mobility programs to the target population and short-term nature of this new program. We also describe how we initiated dialogues with communities, engaged with timely topics, and structured the dialogue throughout the process from program design and pilot program launching to the completion of the pilot program. Fourth, we present the formative evaluation, which focuses on the pilot program’s implementation and process. The formative evaluation results highlight substantial changes for the 10 pilot families, with participants experiencing multi-faceted improvements in mental health, child development, financial security, and income. Finally, the paper concludes with discussion and conclusion sections that offer lessons learned about execution of evaluation processes and recommendations for housing policy.

## Method

### Developmental Evaluation and Formative Evaluation

Michael Patton (2008) classified three purposes of utilization-focused evaluation, defined as “evaluation done for and with specific intended primary users for specific, intended uses” (Patton, 2008, p. 37): developmental, formative, and summative. Developmental evaluation focuses on program development and innovation, while formative evaluation focuses on program improvement, and summative evaluation focuses on judgment of value.

During the early stage of the program design, the purpose of our evaluation was developmental. Among the five developmental purposes classified by Patton (2011, p. 194), our evaluation primarily focused on “adapting effective principles to a local context” and “performative development of a potentially scalable innovation,” as we tailored the principles of housing mobility programs to the target population of our program and considered the relatively short-term nature of this initiative.

In program development, the evaluation team sought to help program designers answer the following questions: How should the Program serve the target population? What aspects of current programs should be adjusted to serve this population with a short-term rental subsidy? How should the Program define the participant criteria? What should be key program elements for this population?

After the program pilot was launched, a formative evaluation was conducted on the Program’s implementation and process. This evaluation was critical for testing and refining the program logic model and design to improve the program. The program evaluation approach was utilized to gauge overall program goals and objectives. This program evaluation sought to answer two basic sets of questions concerning: (1) the process of the program’s implementation and formative adjustments to its processes, and (2) the immediate program outcomes.

The role of evaluators varies depending on the type of evaluation they conduct (Fitzpatrick, 1988; Patton, 2008). In this program, which employed both developmental and formative evaluations, the evaluator’s role was collaborative during the developmental evaluation and more independent during the formative evaluation, albeit the evaluation team met weekly with program implementers to discuss the program’s progress and functioning.

### Research Team Roles and Reflexivity

Given that this study focused on developing and implementing the Program, the research team conducted both developmental and formative evaluations. One of the authors chaired the original steering committee and the national advisory committee for the Program’s pilot and is also a co-founder of the Program. To avoid potential conflicts of interest, this author participated only in the developmental evaluation and did not engage in the formative evaluation. Another author, who also served on the national advisory committee for the Program’s pilot, was more extensively involved in the formative evaluation than in the developmental evaluation. This differentiation of roles was intentional.

The two authors involved in the developmental evaluation approached the Program with initial skepticism regarding its feasibility and potential success. Their critical perspectives helped shape a more reflective and inquiring approach during the Program’s development. For example, they raised concerns such as: Will participants actually move for three years? Is the financial support sufficient to make participation worthwhile? Are social supports in addition to rental assistance really necessary?

To minimize potential bias within the formative evaluation, the research team implemented several strategies. These included clearly defining and communicating the evaluators’ respective roles; employing methodological triangulation (e.g., surveys and interviews); and incorporating multiple perspectives from participants, coaches, and property managers. Additionally, the research team maintained ongoing engagement with peer experts, including the national advisory committee composed of local and national experts in this area. This continuous external input helped the research team understand and interpret evaluation findings and translate insights into programmatic improvements.

### Data Collection and Analysis

This paper presents the design of a housing mobility program and details how we structured the dialogue throughout the process and engaged with various topics based on our developmental and formative evaluations. For the developmental evaluation, we collected three types of data: (1) focus group data from potential participants, (2) roundtable discussions with service providers, and (3) a literature review on program design. We used this information to create a program logic model. Potential participants participated in three focus groups, each lasting approximately two hours: two in December 2016 (each with 11 participants) and one in February 2018 (9 participants). Participants were recruited through neighborhood health centers and community service organizations. Additionally, an all-group roundtable with community stakeholders, including local philanthropy, public sector actors, affordable housing organizations, market-rate apartment owners, and social service providers (over 20 participants) was held in October 2016, followed by two two-hour roundtable discussions with only social service providers in October and November 2016. The all-group meeting was arranged to review progress made in designing the Program and to explore key questions—for example, What does success look like? What if sufficient funding is not raised? and Should the Program require participation in coaching or other training programs as a condition of receiving the rental subsidy? The subsequent roundtable discussions focused on service delivery considerations, including the types of services that should be provided to participants, strategies for coaching and service coordination, and feedback from multiple stakeholders regarding the scope and methods of implementation.

For the formative evaluation, we gathered annual data from surveys and interviews with 10 pilot program participants over a five-year period, spanning from 2018 to 2022, covering approximately four months after relocation to three months after their program exit. Survey, interview, and focus group questions were developed from existing instruments utilized in previous housing mobility and satisfaction studies. Surveys were administered via the Qualtrics online survey system. Interviews with each program participant lasted approximately 30 minutes and were conducted in person, by phone, or online. We also collected interview data from program coaches, participating landlords, and property managers.

For the statistical analysis, we used 2019 survey data, as these data were collected 16 months after the move and included both retrospective and post-move information. Wilcoxon signed-rank tests were used to compare participants’ neighborhood and housing satisfaction and emergency room (ER) usage before (retrospective) and after the move. Neighborhood and housing satisfaction were measured on a 5-point scale: very dissatisfied (1), somewhat dissatisfied (2), neither satisfies nor dissatisfied (3), somewhat satisfied (4), and very satisfied (5). ER usage was measured using six ordered categories (0, 1, 2, 3, 4, and 5 or more), with the “5 or more” category coded as 5 for the analysis. Because the exact number of visits above five was unknown, ER usage was not treated as count data.

Retrospective pre-move data may be subject to response-shift bias (Howard, 1980), as participants may reconstruct their pre-move circumstances based on their situation at the time of the survey (16 months post-move). Therefore, observed pre-post differences should be interpreted with caution.

Additionally, one-sample Wilcoxon signed-rank tests were conducted to assess whether the median response differed from the no-change category (coded as 3) for questions regarding changes in economic and housing circumstances, health, and children’s outcomes after the move. Economic and housing circumstances, as well as income and credit score, were measured on a 5-point scale: much worse (1), worse (2), neither worse nor better (3), better (4), and much better (5). Health and children’s outcomes were measured on a 5-point scale: very negative change (1), negative change (2), no change (3), positive change (4), and very positive change (5). Descriptive statistics, including median ratings and response distributions, were reported to provide context.

Using in-depth qualitative methods, this study comprehensively examined the program design development and program implementation and adjustments before and during the pilot program’s first four years. Reflections from focus group participants were included in the developmental evaluation to capture their needs and wish lists related to community, housing, school, and social support, while reflections from program participants were included in the formative evaluation to provide insights into their experiences with the services. The interviews were analyzed in two iterative stages: open coding and axial coding. During the open coding stage, transcripts were analyzed to identify emerging themes. In the axial coding stage, these themes were refined and integrated to build explanations of the phenomenon.

The original program evaluation activities produced four interim evaluation reports and a final evaluation report (https://familiesflourish.org/reports-and-resources/). The research design of the focus groups with potential participants had expedited review by the authors’ university’s Institutional Review Board. The research design of the program evaluation was reviewed and deemed exempt by them.

## Program Rationale and Description

The Program is built on previous literature about the influence of housing location on life outcomes, challenges to transitioning to and from low- to high-opportunity neighborhoods, and an understanding of the benefits cliff. First, according to previous research, housing location strongly influences education opportunities and lifetime career pathways. Research has shown that children who experience household stability in high-opportunity neighborhoods for even a few years before reaching age 13 have positive long-term outcomes (Chetty et al., 2016). However, some low-income households living in disadvantaged neighborhoods prioritize housing characteristics over neighborhood attributes, which limits their location choice (Wood, 2014). That is, seeking a large affordable home may be possible only in disadvantaged neighborhoods.

Second, HCV holders experience challenges in transitioning from low-opportunity to high-opportunity neighborhoods in a sustained manner due to high rental costs and the cost of paying first and last month’s rent and the security deposit, landlord resistance to accepting vouchers, and limited access to necessary information about these neighborhoods (Kleit et al., 2016). Moreover, at the time of the program was developed, no source of income protections were in effect locally. Source of income protections mandate that a landlord accept payment for rent from any source, such as housing vouchers or alimony. Without source of income protections, it is difficult to use vouchers effectively. Although such protections are now in place locally, challenges with compliance still persist (Gill, 2023).

Third, the benefit cliff, defined as a sudden reduction in federal aid due to a minor increase in earnings, may limit the willingness of households to increase their earnings (Anderson et al., 2022). Another disincentive to income increases is that when an HCV holder’s income rises, the proportion of rent they must pay also increases. To avoid the barriers imposed by the benefit cliff and promote economic improvement, this program is designed with no loss of benefits as incomes rise and stable subsidies regardless of income.

At the time of the Program’s initial development, the metropolitan region was ranked as one of the most economically segregated in the US (Florida & Mellander, 2015), and currently the central city and inner ring suburbs continue to be isolated from community resources such as higher paying jobs, grocery stores, accessible green space. Research also indicates that the metropolitan region was a place where people are unlikely to experience economic mobility if they grew up in highly distressed neighborhoods (Chetty et al., 2022). The rental market is difficult to successfully navigate for people who have traditionally been marginalized from resources, including rental assistance. Moreover, many households qualify for but do not obtain housing assistance; thus, these households continue to live in precarity, experiencing housing and economic instability. Before joining the program, participants generally lived in substandard housing, often in very distressed neighborhoods, and were unable to access housing assistance. Some had experienced episodes of homelessness. Despite being employed, their wages were insufficient to support their families.

Thus, the Program was initiated to address housing stability and affordable housing issues, economic mobility challenges in the metropolitan region, as well as child development needs that neighborhood environment influences. The Program provides temporary rental support in high-opportunity neighborhoods and comprehensive coaching to facilitate the success of individuals and families in their transition to higher-opportunity communities. The Program partners with private sector owners of rental property in high-opportunity areas to create additional sources of affordable, stable housing. These neighborhoods are identified using the Ohio Housing Finance Agency’s (OHFA) Opportunity Index, which provides an overview of opportunity at five quintiles incorporating education, employment, housing, transportation, and health (Kenitzer et al., 2017).

## Foundations of the Program: Early Phases and Team Formation

### Timeline of Program Initial Phase

The process of program design heavily engaged with the target community to understand their potential interest in a limited-term housing mobility program. This engagement occurred in five phases (Figure 1). The *initial discussions* began in early 2016 between two of the co-founders when one—a professional civic leader and realtor—approached another—who was retired affordable housing developer with a law degree. The retired affordable housing developer became the program facilitator during program development and the initial implementation of the program pilot.

**Figure 1.**
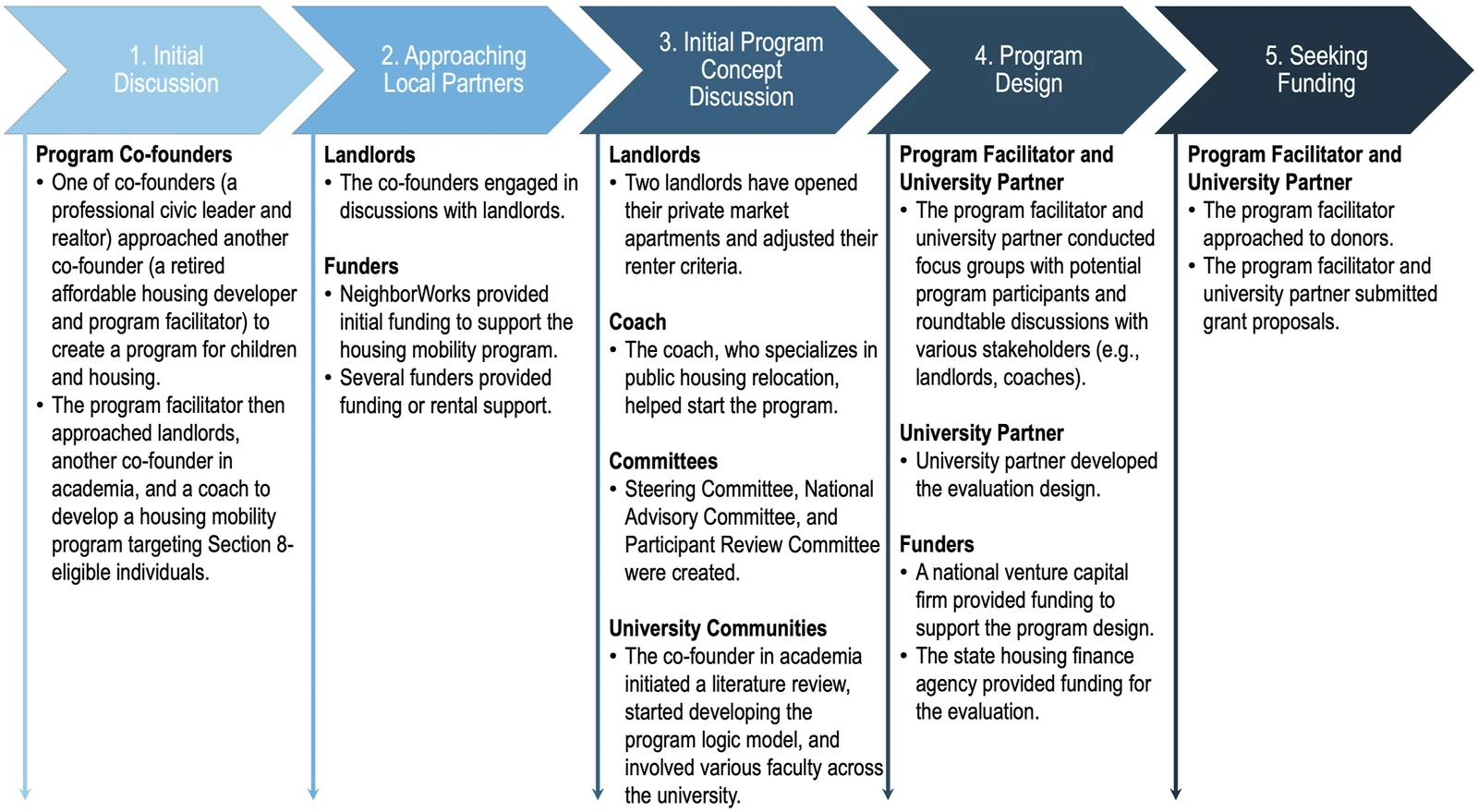
Timeline of program initiation phase

The program facilitator engaged landlords, another co-founder who specializes in housing policy academically, and a coach, to develop a housing mobility program targeting individuals eligible for HCVs. They initiated discussions with various community leaders, which ultimately led to securing commitments from two groups of rental apartment owners in high-opportunity neighborhoods to make their housing available, contingent upon the creation of a program to support the families’ success.

During the second phase, the *approaching local partners* phase, the co-founders engaged in discussions with two landlords and several funders, including NeighborWorks, Ohio Capital Corporation for Housing and the Kelley Family Foundation. They provided funding for rental support.

The third phase was *the initial program concept discussion* phase. The program co-founders along with various individuals working in health insurance, social services, and housing held weekly meetings. These meetings were crucial for making active decisions about program design and evaluation. The co-founders engaged with landlords, the program coach, various committees, and university communities. The landlords opened their private market apartments and adjusted their renter criteria to allow for people with some history of evictions and lower credit scores to rent from them. The program coach, who specializes in public housing relocation, contributed to the coaching curriculum. Several committees were created, such as the Steering Committee (to oversee the program’s functions), the National Advisory Committee (to improve the program and research design), and the Participant Review Committee (to oversee the admission of program participants and trouble shoot if issues arose). The co-founder in academia initiated a literature review, researched current housing mobility programs and local context, started developing the program logic model, and involved faculty from across the university, marking the beginning of our developmental evaluation. Initial potential program design elements were identified through a rigorous literature review of evidence-based best practices from mobility counseling programs.

In the fourth phase, *the program design* phase, the program facilitator and university partner actively made decisions regarding the program design and evaluation. From late 2016 through early 2018, they conducted three focus groups with potential program participants to better understand the participants’ needs. They considered how these needs influenced the criteria for neighborhoods, rental units, and transportation requirements, as well as the participants’ willingness to participate in a limited-term mobility program. Additionally, they held roundtable discussions with service providers and various stakeholders—such as non-profit leaders, landlords, and coaches—to determine the best ways to address participants’ needs. The university partner also developed the evaluation design. A national venture capital firm, through the Ohio State University, provided funding to support program design, and the OHFA provided funding for the upcoming formative evaluation.

Lastly, in phase 5, the program facilitator and the university partner sought funding for the three-year pilot program by hosting fundraisers, meeting with local business foundations and community relations staff, and submitting grant proposals.

### Program Team

The program team was composed of members assigned specific roles: program facilitator, Steering Committee, National Advisory Committee, and university partner. The program facilitator was involved with all aspects of the program, including creating the vision, meeting with people, developing the program, managing regular meetings with the Steering Committee, sporadic meetings with the National Advisory Committee, and other ongoing meetings with various partners. Initially, the university served as the fiscal agent and grant-seeking partner. However, the university was not as effective as a traditional non-profit at a quick turnaround for paying bills. A relationship with a non-profit tax credit syndicator facilitated paying the bills during the developmental period.

The Steering Committee consisted of individuals from diverse backgrounds, including economic, racial, and professional diversity, working in areas such as academia, finance, law, and health. Their role included overseeing all aspects of the pilot, including funding, staffing, compliance with grants and contracts, compliance with the coaching model, and making decisions on participant-related topics (e.g., criteria for program participants, participant selection process, amount of rent support, and location of properties related to the opportunity map) through regular meetings.

The National Advisory Committee, composed of local and national experts, provided input and advice regarding research and program design. This committee included individuals affiliated with institutions such as universities, research institutes, hospitals, health insurance companies, and non-profit organizations.

Additionally, the university partner met weekly with the program facilitator to discuss ad hoc issues related to the program design and the pilot program addressing the program’s implementation process and necessary adjustments, as well as the evaluation process. Lastly, the Program applied for grants through the university as well as receiving a small amount of seed funding from the university.

## Program Design and Development Phase: Developmental Evaluation

We maintained a circular approach to program design, continuously integrating various forms of feedback (Figure 2). This iterative process involved regular circling back to refine the program. Weekly meetings were critical for creating a space for dialogue, troubleshooting, understanding any changes, and maintain constant communication. Preliminary engagement with various stakeholders provided valuable insights and expertise from community members, enhancing the program relevance and effectiveness. Additionally, these engagements offered ideas on how to effectively utilize evaluation throughout the process.

**Figure 2.**
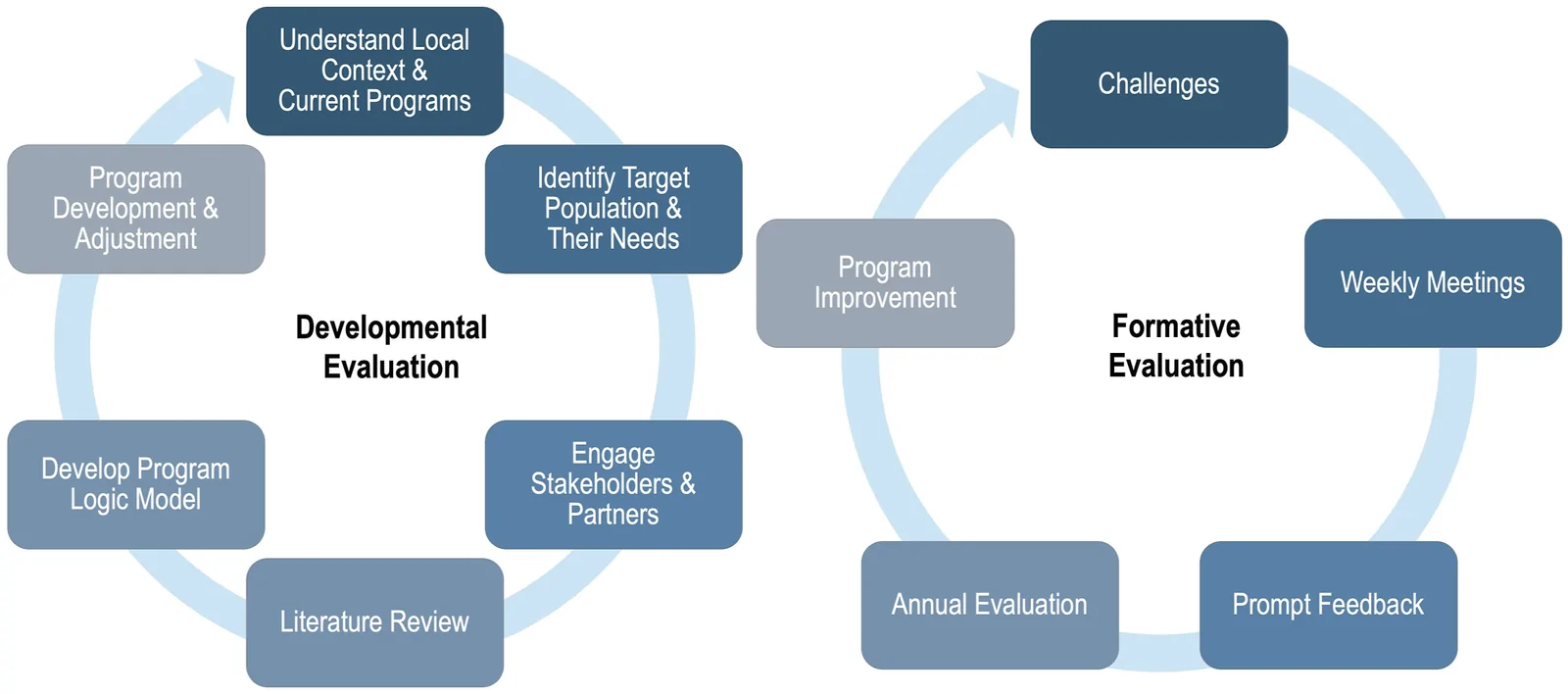
Developmental evaluation and formative evaluation

### Local Context and Politics of Implementation

The Program was created in a time of growing local concern related to the region’s fair housing challenges. The Central Ohio’s growing housing affordability challenges and rates of economic segregation motivated many stakeholders in the region who supported the program. Local media built upon this research focusing a special feature on the extent and impact of economic segregation in the region (The Columbus Dispatch, 2017). Research emerging from Raj Chetty’s research team at Opportunity Insights raised the profile of the issue of economic segregation in relation to child development needs in the community (Chetty & Hendren, 2018).

The growing housing affordability crisis in the region motivated organizations across the city and county to explore new affordable housing models. The Affordable Housing Alliance of Central Ohio was formed as they worked on a 2017 study that documented increasing affordability problems and unprecedented eviction rates in the region (Affordable Housing Alliance Central Ohio, 2017). The 2017 study found the central county of the metropolitan region to be experiencing a shortage of more than 50,000 affordable rental units. Housing affordability and instability was particularly challenging for the communities low to moderate income renters (Affordable Housing Alliance Central Ohio, 2017).

The Program was supported by a wide and diverse group of stakeholders that ranged across the political spectrum. Liberal stakeholders were attracted to the program’s ability to expand affordable housing options. Conservative stakeholders were attracted to the program’s focus on family economic self-sufficiency. Program volunteers were often successful professionals who had navigated childhood experiences in poverty. Several large rental apartment companies supported the program as a form of philanthropy. The local real estate association supported the program as a regional fair housing solution.

The Program did face a number of obstacles to implementation due to uneven local political support. The state’s housing finance agency supported the program financially, and the governor’s office supported it politically, but local public sector backing was very limited. Initially, neither the central city or local county governments, nor the local housing authority supported the Program.^2^ Opposition from local government at that time focused primarily on concerns related to moving participants outside of city boundaries. Additionally, concerns about undermining ongoing neighborhood revitalization efforts did not come to fruition. The local chapter of the NAACP was also concerned about the program’s impact on the central city’s school district and the likelihood of families of color facing racial discrimination in their new neighborhoods.

In a highly competitive funding environment for affordable housing dollars, the program required a non-traditional funding structure. Instead of traditional public sector support, supplemented by philanthropic giving, the Program was primarily funded through local philanthropic giving, often at the individual level. Individual donors were highly motivated to support the program because of its direct impact on families.

### Target Group and Needs Assessment

#### Identifying the Target Group: Eligibility Criteria and Rationale

The pilot program focused for several reasons on very low-income single-mother households living in challenging environments who did not obtain an HCV. First, housing affordability and instability are highly gendered issues in this community; very low-income single-mother households comprise the majority of low-income families in the region. Second, this target population was experiencing an increase in homelessness.

Based on the program’s target population, the Steering Committee discussed the specific criteria for participant selection. Eligible pilot applicants were very low-income single female-headed households with adults at least 18 years old. The participant families had 1 to 3 children, ages 13 and under, with at least one child in school and eligible for Medicaid. Household income was between 30% and 50% of the area median income based on family size ($23,000–$37,000). They must not have been current participants in the HCV program. They lived in low-opportunity areas before participating in the program and had access to transportation. Additionally, they were willing to meet monthly with a coach, attend monthly programs, and move to a community connected to the program.

#### Needs of the Target Group

Through three focus groups, potential participants shared their needs and wish lists across various aspects of the program—specifically the suburban rental housing program—such as their desired community, apartment, school, and social support. Their primary concern was neighborhood safety, and they expressed a desire to live in suburban areas with more resources. They wanted to live in areas with less crime, access to bus stops, activities and events for young children, outdoor play opportunities, community amenities, and nearby fitness centers. Here are some reflections from the focus groups with potential participants regarding the neighborhood environment:

“I think the school districts is [sic] definitely an attraction for me. And just the diversity. I mean it’s diverse.”

“… I’m looking for a great school district, less crime area. [Central city] has a lot of crime.”

“I would prefer to live in a community where we’re doing stuff every week, every weekend, every Friday, Saturday, where there’s a movie outside …”

Similarly, the focus group participants listed their preferred apartment features, including laundry hook-ups in the unit, central air conditioning, hardwood floors, upgraded appliances, open space, ample closets, spacious apartment, patios or backyards, garages, large kitchens and bathrooms, 24-hour maintenance, and 2 to 3 bedrooms.

They wanted neighborhoods with some diversity and little racial bias. Regarding schools, they wanted teachers to be strict about bullying, to show care and interact with parents, to offer better teaching and education levels, to have highly rated schools, to provide diversity, to give individual attention to kids, and to have sports available. The following is a selected reflection from the focus groups with potential participants on educational quality:

“I’m just saying my kids, growing up, from [central city’s school] and academy school with that … she was making honor roll. My daughter was an honor roll student. And now since she’s in [suburban city] and she’s [in a higher grade], you’re calling and telling me … it’s second quarter … second school quarter now … and now you’re telling me my daughter is at a [lower] grade level reading. That’s not right.”

The focus group participants expressed a need for assistance with financial and personal matters, such as credit counseling, help with planning to purchase a home and saving for a down payment, employment planning and finding new jobs, steps to achieve these goals, support for children’s school needs, information about the new community, a stable place where they can stay for all three years, a comfortable living environment, and assistance with moving toward self-sufficiency. Below are selected reflections from the focus groups with potential participants:

“Another thing, for me, would be like to have … like for if you got into this … although the program is only three years—right?—are we going to have tools to help set us up to move, like maybe some credit counseling?”

“But during these three years I would like to … with the person that will be assisting us, if this does go through, I would like to sit down and go over my finances and how to better myself within the next three years, whether it is if I want to stay here or whether I want to go somewhere else.”

They also shared perspectives regarding the proposed communities as potential program locations, assessing their suitability. Some participants were interested in relocating to suburban areas but reflected on whether such a move would be practical given the distance from their familiar surroundings. Additionally, several participants noted their limited familiarity with these communities, as they are not within their usual travel routes or workplaces. This prompted program design considerations about extended commutes, the distance from their current residences, and how well they might integrate into the new environment. Participants also expressed concern about the potential academic adjustment for their children, given that the new school may have more challenging academic standards.

Through this conversation about the lack of familiarity with different schools and neighborhoods and concerns with financial and personal matters, it became apparent that many focus group participants had little experience with many of the things about which they were asked and had little knowledge of where to go for help. While they thought the communities proposed were very attractive and they would like their children to attend school in suburban communities, the cost of living was too high. Additionally, they had concerns about the racial diversity of the communities and whether or not they would feel at home. Their perspectives were based on the information they had heard about the communities and not on first-hand knowledge or experience. Such information usually is gleaned from the social ties people have—their social capital. However, social capital can also be limiting, in that information available through social ties is only as good as the information within that social circle (Portes, 1998; Portes & Landolt, 1996). And if the trusted social circle has no experience with new places or ideas, often it is hard to learn about them. Combination of these findings with this literature led the integration of personal coaching into the program to help participants take and act upon new information about places, ideas, and practices.

### Initial Developmental Model

An initial developmental model, the predecessor of the program logic model, summarized the anticipated outcomes and provided a comprehensive strategy (see Table 1). According to the W. K. Kellogg Foundation (2004, p. 1), a logic model is defined as “a systematic and visual way to present and share your understanding of the relationships among the resources you have to operate your program, the activities you plan, and the changes or results you hope to achieve.” This initial developmental model is thus grounded in a “theory logic model,” which underscores “the theory of change that has influenced the design and plan for the program” (W. K. Kellogg Foundation, 2004, p. 9). To develop the program effectively, we needed to understand and explain why it was needed and identify the components that would be effective in its creation. This model clarified our expectations and served as a tool to present the program’s theory of change and expected results.

**Table 1.** Initial Developmental Model

​	Beginnings	Planned work		Intended results		
	Assumptions	Inputs	Activities	Outputs	Outcomes	Impacts
Households & subsidy	Regional affordable housing shortage: There are more than 40,000 severely housing cost burdened households	Households	Establish willingness to relocate	Relocate to affordable and stable housing	Increased short-term housing affordability and stability	Increased housing affordability and stability
	Low-income households below 80% AMI qualify for Section 8; there are more than 25,000 households on the Section 8 waitlist	Public housing authority	Utilize list of Section 8 eligible population	Target population	Reduction of Section 8 waitlist; temporary increase in number of affordable housing units	Decreased demand for Section 8 affordable housing units
	High costs of rental housing prohibit very low-income households between 30% and 50% AMI from renting in high-opportunity neighborhoods; high costs of rental housing lead to housing instability	Funders	Provide affordable housing subsidy in high-opportunity neighborhoods	Relocate households to affordable housing in high-opportunity neighborhoods	Increased short-term opportunity for relocating households	Increased economic opportunity and mobility
Social supports	Built into the cost of high-opportunity neighborhoods are local schools and school districts; households with children under the age of 14 are most affected by school choice; many central-city school’s state ratings are among the worst in the region, while many suburban schools rank among the best.	Suburban schools	Enroll students in suburban school districts	Attendance in suburban school districts	Decrease in absences; Increase in test scores	Increase household educational opportunity and mobility
	Households in low-opportunity neighborhoods are exposed to high levels of emotional, environmental, financial, intellectual, occupational, physical, social, and spiritual stress	Wellness support services	Provide wellness support	Residents move from low-opportunity neighborhoods to high-opportunity neighborhoods with appropriate support services	Reduction in stress; increase in personal and household wellness (ex. Increased income, decreased health costs)	Increased wellness, and household independence
	In extreme instances, households in low-opportunity neighborhoods are affected by trauma	Trauma support services	Provide trauma support		Reduction in trauma exposure; healthcare savings	
Housing	Landlords are less willing to rent to applicants who have poor credit, negative rental history, inadequate income or provide incomplete information	Landlords w/apartment developments in high-opportunity neighborhoods	Revise and update leasing requirements	Creation of new leasing agreements; acquisition of lease agreements	Lower turnover costs; Increased tenant retention	Reduction of barriers to opportunity
	Apartment complex management, staff, and maintenance crews frequently interact with residents, and may stigmatize them because they have lower socio-economic status or rental assistance; the residents may not blend in socially; reducing potential stigma is critical to demonstration project success	Staff at apartment developments in high-opportunity neighborhoods	Initiate and complete implicit bias training	Increased staff awareness of how implicit bias impacts housing	Improved staff-tenant relations	
Development & research	Local, regional, and national stakeholders are interested in the intersectionality of affordable housing, education, wellness, trauma, and implicit bias	Stakeholder consortium	Support and externally advise demonstration project	Steering committee; advisory documentation; contacts; funding sources	Increased knowledge of demonstration effectiveness; savings regarding service provision	Model affordable housing policy
	Developing a demonstration program requires an organization to guide the development of the demonstration program	Steering committee	Internally advise and guide demonstration project program protocol, implementation, assessment, and evaluation	Demonstration project organization; project protocol; disbursement of subsidy through enabled fiscal agent	Successful direction of demonstration project; development of replicable demonstration model	
	To advise, monitor, and assess the demonstration program, a diverse array of experts provide leading-edge thought	Research hub(s)	Internally assemble research hub(s) to develop, monitor, assess, and evaluate the demonstration project	Literature review(s); monitoring, assessment, and evaluation of demonstration project; research reports, papers, and books	Knowledge summarization and creation	

*Note.* Adapted from the W.K. Kellogg Foundation’s example of a Theory Logic model (2004, p. 11).

The initial developmental model presents in great detail all the assumptions, inputs, activities, outputs, outcomes, and impacts of the program prior to its implementation, as adapted from the W.K. Kellogg Foundation’s work (2004). The following definitions are also adapted from this framework. The assumptions outline the rationale for the program’s necessity and effectiveness, grounded in the local context, literature, and data. Inputs refer to the resources necessary for program implementation. Activities encompass the processes and actions required to implement the program and achieve its goals. Outputs are the immediate, measurable results of program activities. Outcomes represent the short- and long-term changes resulting from these activities, including shifts in behaviors, or status. Impacts reflect the broader, long-term changes that include individual-level improvements and changes at the community or policy level, such as improved conditions or shifts in policies. This model consists of four key sections: households and subsidy, social supports, housing, and development and research.

The first horizontal section, *households and subsidy*, includes three assumptions. The first assumption is that the region has a shortage of affordable housing, with over 40,000 households experiencing severe housing cost burdens. Successful program implementation requires households that demonstrate a willingness to relocate. Once the program activities are in place, participating households will relocate to affordable and stable housing, leading to short-term improvements in housing affordability and stability. Over time, this will contribute to increased housing affordability and stability among the population served.

The second assumption is that low-income households earning below 80% of the area median income qualify for HCVs, with more than 25,000 households on the HCV waitlist at the time of the study. The local public housing authority maintains that list, and the hope was to reduce the waitlist by housing the same target population. Over time, the program would contribute to a decreased demand for HCVs in the community.

The third assumption is that the high cost of rental housing prohibits very low-income households earning between 30% and 50% of the area median income from renting in high-opportunity neighborhoods. Additionally, these high rental costs contribute to housing instability. Successful program implementation requires funders to provide subsidies for affordable housing in these high-opportunity neighborhoods. Once the program activities are in place, participating households would relocate to affordable housing in high-opportunity neighborhoods, resulting in immediate increased access to opportunities for those households. Over time, this would lead to greater economic opportunity and mobility for participating households.

The second row, *social supports,* comprises three assumptions. The first assumption is that bundled into the cost of housing in high-opportunity neighborhoods is access to high-performing local schools and school districts. Additionally, households with children under the age of 14 are most affected by school choice. Many central-city school’s state ratings are among the worst in the region, while many suburban schools rank among the best in state ratings. Program implementation requires suburban schools to enroll students from participating households. Once the program activities are in place, these students will attend suburban schools, resulting in decreased absenteeism and improved test scores. Over time, this will contribute to greater educational opportunity and mobility for participating households.

The second assumption is that households in low-opportunity neighborhoods experience high levels of emotional, environmental, financial, intellectual, occupational, physical, social, and spiritual stress. Program implementation requires wellness support services to provide comprehensive assistance. Once the program activities are in place, residents will move from low-opportunity neighborhoods to high-opportunity neighborhoods with access to appropriate support services. This transition will lead to a reduction in stress and an increase in personal and household wellness (e.g., increased income and decreased health costs). Over time, this will contribute to enhanced wellness and greater household independence.

The third assumption is that, in extreme cases, households in low-opportunity neighborhoods are affected by trauma. Program implementation requires trauma support services to offer necessary care. Once the program activities are in place, residents will move from low-opportunity neighborhoods to high-opportunity neighborhoods with access to appropriate support services. This transition will lead to reduced trauma exposure and increased healthcare savings. Over time, this will contribute to improved wellness and greater household independence.

The third section, *housing*, involves two assumptions. The first assumption is that landlords are less likely to rent to applicants with poor credit, negative rental histories, inadequate income, or incomplete application information. Successful program implementation requires landlords who own rental properties in high-opportunity neighborhoods to revise and update their leasing requirements to better accommodate these applicants. Once the program activities are in place, the following outputs will be achieved: the creation of new leasing agreements and the acquisition of lease agreements. These changes will result in lower turnover costs and increased tenant retention. Over time, this will contribute to a reduction in barriers to housing opportunity.

The second assumption is that apartment complex management, staff, and maintenance crews frequently interact with residents, and may stigmatize them because they have lower socio-economic status or rental assistance. The residents may not blend in socially. To reduce potential stigma toward program participants, program implementation requires staff at rental properties in high-opportunity neighborhoods initiate and complete implicit bias training. Once the program activities are in place, staff awareness of how implicit bias impacts housing will increase, leading to improved staff-tenant relations. Over time, this will contribute to a reduction in barriers to housing opportunity.

The final horizontal section, *development and research*, involves three assumptions. The first assumption is that local, regional, and national stakeholders are interested in the intersection of affordable housing, education, wellness, trauma, and implicit bias. Program implementation requires a consortium of stakeholders to provide support and external advisory for the pilot project. Once the program activities are in place, several outputs will be achieved, including the establishment of a Steering Committee, advisory documentation, contacts, and funding sources. These outputs will lead to increased knowledge of the pilot’s effectiveness and cost savings in service provision. Over time, this will contribute to the development of a model for affordable housing policy.

The second assumption is that developing a pilot program requires an organization to oversee its development. Program implementation requires a Steering Committee to provide internal advice and guide the pilot program’s protocol, implementation, assessment, and evaluation. Once the program activities are in place, the following outputs will be achieved: the organization of the pilot project, the project protocol, and the disbursement of subsidies through an enabled fiscal agent. These outputs will lead to the effective management of the pilot project and the creation of a replicable model. Over time, this will contribute to the development of a model for affordable housing policy.

The third assumption is that a diverse array of experts provides leading-edge thought to advise, monitor, and assess the pilot program. Program implementation requires a research hub to assemble research teams internally to develop, monitor, assess, and evaluate the pilot project. Once the program activities are in place, the following outputs will be achieved: literature reviews; monitoring, assessment, and evaluation of the pilot project; and research reports, papers, and books. These outputs will lead to the summarization and creation of knowledge. Over time, this will contribute to the development of a model for affordable housing policy.

Through formative evaluation during the pilot phase, the initial developmental model was refined into the program’s logic model (see the “Pilot Program Outcomes Phase” section). The following discussion of program components and implementation outlines the transition from the ideas in the developmental model to the program’s logic model.

### Key Components of Program

Using the Initial Developmental Model and the literature, the program team considered additional barriers to program participant success and created program components to address them.

#### Overcoming Structural Barriers

Our literature review and understanding of the local context and politics helped us shape the program. The target population in this program faced several structural barriers, including exclusionary land use policies, a limited supply of affordable housing, and socio-economic precarity. To address these barriers, the pilot program employed a multi-faceted intervention that included: a privately funded $400 monthly rental subsidy, access to high-quality rental units in high-resourced and safe neighborhoods, monthly programs, and life coaching over a three-year period.

#### Move Locations

To meet potential participants’ needs regarding community aspects such as good school districts and lower crime rates, the Steering Committee discussed the use of OHFA’s Opportunity Index to determine whether the program should include high-opportunity areas. The location of the properties included multiple suburban jurisdictions in the region which offer opportunities for safe neighborhoods, high-performing schools, and employment. Additionally, the Steering Committee discussed whether the program should require children to attend public schools in their new community. In the end, the program chose housing in high-opportunity areas and mandated that children go to schools in the districts associated with that housing.

#### Landlords and Program Support

Two landlords participated in the pilot program by providing 10 apartments in high-opportunity areas and additional rent support of $100 per participant per month, supplementing the amount the program provided. Importantly, the landlords revised their leasing requirements to accept “challenged” applicants who they would usually reject due to inadequate income, poor credit score, eviction record, negative rental history, debt, and other background concerns. By participating in the program, landlords benefited from lower turnover costs and increased tenant retention.

To support participants and remove barriers to high-opportunity communities, the program offers implicit bias training for property management personnel. Since apartment complex management and staff frequently interact with residents, and the staff may know of the tenant’s involvement in the program, they might stand out to staff. This training aims to improve relationships between staff and tenants by overcoming implicit bias on both sides.

The participating landlords and property management personnel provided ongoing input regarding participant-related concerns on their properties. They contacted the program facilitator to discuss any issues regarding the pilot or their tenants.

#### Life Coaching and Monthly Programs/Workshops

Unlike previous housing mobility programs, the Program incorporates life coaching and monthly programs/workshops, alongside rental support and relocation to high-opportunity areas, to address the unique challenges participants face. In a limited-term program, participants are keenly aware that their rental assistance will end and use the three years to plan accordingly. Focus groups with potential participants revealed that many participants may have limited experience and social capital, which may hinder their ability to navigate the changes associated with moving to a new community—such as identifying resources for assistance or adapting to unfamiliar environments. In response, we designed the Program drawing on evidence-based best practices, including not only mobility counseling programs (Darrah & DeLuca, 2014) but also public housing authority self-sufficiency programs (Brennan, 2014; Kleit, 2004; Rohe & Kleit, 1997, 1999) and financial coaching programs (NeighborWorks America, 2014).

To facilitate a successful adjustment, the program emphasizes the establishment of trusted relationships that enable participants to absorb and act on new information regarding their surroundings, available opportunities, and local resources. This rationale guides the integration of one-on-one coaching and monthly programs into the program’s design. Furthermore, life coaching and monthly programs/workshops focus on four pillars: housing stability, financial capability/stability, improved health outcomes, and education/career development. These components are designed to support participants in critical areas and ensure they have the tools and support needed to thrive in their new environments.

## Pilot Program Implementation Phase: Formative Evaluation

In March 2018, a three-year pilot program was launched to test program processes, assess program sustainability, document initial program impacts, and refine evaluation protocols before moving into full implementation. Out of over 300 applicants, 10 participants were selected and began mobility coaching in late spring 2018, followed by relocation to high-opportunity neighborhoods in summer 2018. They also started participating in coaching and monthly programs. Regularly they participated in the program evaluation.

One family exited in November 2018 when they moved to another state, and another family joined in December 2018 to replace them. In May 2020, the pilot was extended for a fourth year due to the COVID-19 pandemic; Steering Committee discussions and participant focus groups informed the decision. One family exited the program in the summer of 2020 because the participant thought she was capable of being self-sufficient, while nine families continued in the fourth year. The pilot concluded at the end of July 2022.

The Program was largely implemented as planned; however, as a newly developed initiative, adjustments were necessary to support participants in various ways. For example, the program established partnerships with several organizations to offer specific career training and child mentoring programs.

### Challenges and Solutions

During the pilot period, the program encountered several challenges and developed solutions through weekly Program team meetings, prompt feedback, and annual evaluations. These occasions provided ideas for program revisions for the pilot program and a subsequent demonstration project.

#### Insights From Initial Results and Rapid Feedback

The first significant challenge was coach transitions. A few months after the program started, the original coach left, prompting a transition from a single coach to a networked coaching system. The networked system would provide back-ups in case something happened to a coach. This change required participants to rebuild trust with their new coaches. Interim coaches worked with participants during the transition, and four new coaches and a coaching coordinator joined the program several months after the original coach’s departure. The new coaches each came from a professional life-coaching background and had diverse professional experiences; each worked with two participants. They brought their unique expertise and networks to the coaching relationship. Among the coaching team was a financial specialist who provided support for the more complex financial challenges faced by the participants. The coaching coordinator supported the coaches and provided support when issues required social work expertise instead of coaching.

Although some participants expressed concerns during the initial coaching transition, six months into the new coaching system, feedback indicated that participants were generally very satisfied with the new coaches and coaching model. The relationship-building period with the new coaches and the alignment between the coaches’ expertise and participants’ goals facilitated a smooth transition. The program learned from this experience, which provided a foundational understanding for the future demonstration project on how to manage multiple coaches. Here are some reflections from the interviews with program participants regarding the coaching transition:

“It was a little difficult in flux when we had that transition. I’m really happy with the coach that they paired me with. She’s been absolutely wonderful. She’s been really helpful in helping me reprioritize things and it’s going great.”

“I’m starting to open up to her more, taking what she’s giving me, not being like I’m here just because I’m here. She’s here because she cares. It’s just letting her in and me just day to day like learning. I’m trying to figure out who I am.”

“Everybody likes their coach. It’s almost like they were almost handpicked for us. What our interests are, hobbies, help navigate through community resources, or what’s out there that maybe can be utilized through any of us or information to pass on to others.”

The second challenge was the impact of the COVID-19 pandemic. The pilot was extended for one year because the pandemic deeply affected many participants and their families. In response to the pandemic, the monthly programs and coaching sessions shifted from in-person meetings to virtual meetings, for which the program provided Chromebooks to the participants who expressed a need for them. The participant families navigated disruptions to employment and income, adapted to online schooling for their children, and faced deep concerns about their families’ health. The following are selected reflections from interviews with program participants on the pandemic and program support:

“The programs taught me how to budget. They taught me how to save. They helped me learn healthy habits that literally changed what could have been a very scary outcome for my family. The pandemic would have really set us back if I hadn’t learned these skills from the program which better prepared us for the unexpected.”

“I never had a budget set before, without my budget I would have drown [sic]. Over the last 2 years I’ve been able to establish an emergency fund which I’ve had to tap into, I am so happy to have had it in place.”

“Having someone to listen, bounce ideas and strategies off of, help me remember to check the pros and cons was, I feel, invaluable during a sudden time of uncertainty in the world. She kept me grounded and cheered me on!”

“(I benefited from) Mindfulness and resources for pandemic stress relief.”

“I’m much happier where I live now, although the pandemic took its toll on all of us, I’m not in a home that’s barely standing or overwhelmed with outrageous utility bills. I can get the kid outside in a safe environment and take a walk and explore nature a little bit. There is a lot of places to get out and safely get a breath of fresh air while social distancing.”

“We are in a safer and healthier environment all around. I feel our current environment made it easier to quarantine.”

A third challenge was a few issues with bullying and concerns about discrimination. While several participants reported freedom from bullying in their new school, two participants mentioned that their children were being bullied. One family, with the assistance of program staff and the participating landlord, relocated to another school district, which remedied the situation. The other family did not change schools and worked with the Program and the school to resolve the issue.

#### Unexpected Outcomes

One unexpected outcome was early program exiting. One participant left the program about 19 months after joining because she believed she was ready to stand on her own. This was unforeseen before the program launch. The program team was curious whether the three-year rental subsidies and comprehensive coaching would assist participants in achieving self-sufficiency after program completion. This experience gave the team some confidence that the Program was on the right track.

Another challenge was a significant increase in rents in the regional metropolitan housing market. The team thought this could become problematic after the program ended, as participants would have to pay rent without any assistance. Upon completion of the program, landlords provided a one-year rent freeze for participants. Based on exit interviews, most participants were able to handle the absence of rental support and remained in the same apartment complex they lived in during their participation in the program, which exceeded expectations.

#### Raising Financial Support

Since this is not a federally funded program, a major challenge has been raising funds for program implementation. Local funders provided funding for planning grants, and the state’s housing finance agency provided funding for program evaluation. As a new program without a track record it was difficult to secure financial support from many funders. Outreach to individuals within the community eventually proved to be the most effective fund-raising strategy. Individuals supported the Program because they themselves grew up in poverty and wanted to give back, others donated because they felt that the contribution would lead to the sort of outcomes for families facing economic hardship that they would like to see. Eventually, the program commenced after raising 70% of the three-year budget and securing enough funding to cover rental assistance for 3 years. In addition, the program was able to raise resources for updated budget for a one-year extension following the COVID-19 pandemic.

## Pilot Program Outcomes Phase: Formative Evaluation

### Overall Program Effectiveness

The formative evaluation offers valuable insight into participants’ experiences and perceived benefits across various areas, based on surveys and interviews, although the evaluation was descriptive in nature and not structured to establish causality. Program participants reported improvements in their residential and economic stability over the course of the program; most remained in the same apartment complex in higher-opportunity neighborhoods without additional assistance and saw improvements in their income during the Program. Through comprehensive life coaching and housing support in higher-opportunity neighborhoods, the program helped participants relocate to high-opportunity areas for three years and supported them in setting and working toward personal goals, such as financial and employment objectives. Findings from the pilot suggest that participants experienced outcomes that exceeded initial projections in areas such as housing and financial stability, improved health outcomes, and educational and career advancements.

Wilcoxon signed-rank tests were used to compare participants’ neighborhood and housing satisfaction before (retrospective) and after the move. Results indicated a significant increase in satisfaction after the move regarding neighborhood safety, cleanliness, police protection, convenience to shopping, and housing quality (Table 2). We also used a Wilcoxon signed-rank test to compare participants’ ER usage before (retrospective) and after the move. Results indicated a significant decrease in ER usage following the move (Z = 2.156, *r* = 0.6818, p = .0311).

**Table 2.** Wilcoxon signed-rank test results for neighborhood and housing satisfaction before (retrospective) and after the program participation and relocation (*n* = 9)

	Wilcoxon Z	*r*	p-value
Neighborhood safety	−2.661	−0.8870	.0078
Neighborhood cleanliness	−2.695	−0.8983	.0070
Police protection	−2.373	−0.7910	.0176
Convenience to shopping	−2.219	−0.7397	.0265
Housing quality	−2.668	−0.8893	.0076

*Note.* One participant did not provide responses for new neighborhood satisfaction across all aspects. Effect size (*r*) represents the rank-biserial correlation calculated as (Z/√N). To account for multiple comparisons across the 13 total Wilcoxon signed-rank tests conducted in this study, p-values were adjusted using the Benjamini-Hochberg False Discovery Rate (FDR) procedure. All results with a raw p < .05 successfully survived this correction threshold (q < .05).

Table 3 presents descriptive statistics and one-sample Wilcoxon signed-rank test results for post-move changes across key outcomes. Participants reported generally neutral to positive changes across domains, with statistically significant shifts from the no-change category after the move, indicating overall positive changes in economic, income, credit score, housing, mental health, children’s outcomes.

**Table 3.** Descriptive statistics and one-sample Wilcoxon signed-rank test results for post-move changes across key outcomes (16-month post-relocation)

​	*n*	Median	% Negative (1−2)	% Neutral (3)	% Positive (4−5)	Wilcoxon Z	*r*	p-value
Economic circumstances	10	4.5	0	10	90	2.811	0.8889	.0049
Income	10	4	0	10	90	2.811	0.8889	.0049
Credit score	10	4	0	20	80	2.828	0.8943	.0047
Housing circumstances	10	5	0	0	100	2.889	0.9136	.0039
Mental health	10	4	0	30	70	2.568	0.8121	.0102
Children’s behavior change	9	4	0	11.11	88.88	2.658	0.8860	.0079
Children’s overall health	10	4.5	0	20	80	2.711	0.8573	.0067

*Note. n* varies due to item-level missing data; one participant did not provide responses for the children’s behavior change item. Effect size (*r*) represents the rank-biserial correlation calculated as (Z/√N). To account for multiple comparisons across the 13 total Wilcoxon signed-rank tests conducted in this study, p-values were adjusted using the Benjamini-Hochberg False Discovery Rate (FDR) procedure. All results with a raw p < .05 successfully survived this correction threshold (q < .05).

While the calculated rank-biserial correlations indicate large effect sizes across our significant variables, caution should be exercised when generalizing these findings because of the limited sample size (*n* = 9 or 10).

### Program Outcomes

The evaluation followed specific program outcomes, incorporating perspectives from both participants and key stakeholders (e.g., landlords, property managers, coaches). First, most participants reported that their current neighborhood was much better than the one they lived in before joining the program, according to the program exit survey conducted a few months after leaving the program. The majority of participants reported improvements in economic and housing circumstances, physical and mental health, and children’s academic and health outcomes. Analyses presented in Table 3 indicate statistically significant positive shifts in participants’ reported income and credit scores after the move. Consistent with these results, program administrative data showed an average income increase of 58%, and a 22% growth in credit scores during the four-year pilot period. Additionally, children’s respiratory health appeared to improve, and the number of ER visits declined. Here are some reflections from the interviews with program participants regarding the neighborhood environment, economic circumstances, health, and children’s outcomes:

“[in the old neighborhood] drugs, gangs, and worrying about the kids being influenced by some of the other kids, not making good choices.”

“It’s really really nice. Just being in a place where I know it’s healthy for my kids and they’re safe and I don’t have to worry, all that stress and anxiety is no longer an issue. That has definitely made a really big difference.”

“We have just gotten more and more settled here in the area. Just really feeling a part of the community. I even recently had a job transfer to “my” location here, so it just makes it feel even more like home.”

“I’m saving more money now, because I am not paying as much [for rent] it takes a lot of stress off.”

“I am at the same employer and have received several promotions.”

“It [my income] has nearly doubled.”

“My stress level has gone down tremendously. Like I said I love just letting my kids go outside and play and I don’t have to worry about her playing in the backyard like before.”

“…I realized that I wasn’t running to the emergency room for asthma treatments. It was so much of a difference to me. But we haven’t had those same issues because of where we are and the quality of the apartment.”

“My son’s asthma is really doing well now. This is where we’re supposed to be now.”

“…she’s doing really good as far as her grades. She’s doing really really well. She’s having a lot of new friends and meeting people. She’s not being teased as much. She’s also reading above grade level I found out.”

“She’s been doing really well. She tested gifted in a few categories…we’ve started [the current] grade now and she’s kind of just cruising right along.”

Second, key stakeholders were positive about the pilot program and participants. Two landlords indicated that they would recommend participation in the program to other property owners. Additionally, they agreed that the program’s stable tenants provided financial benefits, including cost savings from reduced annual turnover and marketing expenses. Most property managers reported positive interactions with participants and stated they were very or somewhat likely to recommend participating in the program to other property managers.

Third, five program coaches from various professional backgrounds worked with two participants each. Based on interviews, they indicated that their overall experience of the program had been positive and that they observed positive changes in the participants. Below are selected reflections from interviews with the program coaches:

“It has been very rewarding to see the impact this program has on the moms and the kids.”

“Glad to be part of the program and the opportunity to work with the moms…help them to achieve their goals—awesome!”

“It has been positive and supportive experience. Luckily, we had established trust and a good base so communicating deep feelings was enabled.”

“Fulfilling. Meaningful.”

“Two of my moms have been quite successful—pursuing dream jobs and have made progress in meeting their financial goals.”

Fourth, most participants rated their overall experience in the program as positive. In interviews, they consistently described positive changes in housing quality and neighborhood safety as key aspects of their new housing situations. Additionally, most participants characterized the coaching and monthly program components as positive or very positive. When asked which elements they found most beneficial, participants’ responses varied, highlighting rent support, stable and healthy housing in safe neighborhoods, coaching, monthly programs, and peer group interactions. Below are selected reflections from the interviews with the program participants on neighborhood environment, housing quality, coaching, monthly programs, and overall experiences:

“The difference is night and day. My old area was becoming unsafe. We did not feel comfortable sitting on the porch at night. We [now] have a patio we can sit out on in the evening and enjoy it. We feel safe.”

“Health!!! Safety!!! Condition!!! Landlord/rental staff!!! In every way BETTER!”

“Coaching has been really helpful. Very insightful…kind of gives me a foundation to build on so that I can be successful and probability to stay where I am at.”

“Very positive! I learn something new every month! The meetings really keep me going!”

“Getting the information is always nice and then being able to sit with nine other women who are all smart and have different personalities. We all have different things to bring to the table.”

“I feel like I am seeing personal growth within myself and my daughter. She has improved a lot as far as being acclimated to her new home, the whole new school district, the teacher and with me. I’m just learning a lot, trying to get into making more money. Just the way I think now is a lot better. I grocery shop a little different. I’m not exactly where I want to be, but I still have work to do and that is okay. I’m better off than where I was.”

Fifth, participants shared the following reflections in the exit interviews (selected examples are shown below):

“I definitely had an improvement. I didn’t have negative anything. Anything within it definitely benefited the school change, kid’s attitude, my attitude, just a way to view different people that I meet from different nationalities.”

“I’m so thankful to you all for this program and all the opportunities that we’ve had, that we wouldn’t have otherwise had. We love our neighborhood and our home and the school district, and just everything’s been so great. I’m just feel really grateful and really thankful for everyone that was able to make this happen.”

“It [Program] just allows me to restart, close off one chapter, and start anew, so (this is) my fresh ground. So yes, it was the best thing that happened to me.” “It definitely was a great experience. I learned a lot about myself over the years. Confidence level has changed. Anxiety level has changed. So, everything got better. So yes, it’s a good program.”

“I will say I’m extremely comfortable. … I know I have some money and savings, not worrying paycheck to paycheck, so it’s not perfect, but I’m able to feed them.”

“…I knew I needed to get to a better area. I knew that I needed to get my daughter into a better school district. I never imagined that I would have all of this. I never imagined that the hospital visits would stop, that our overall health would improve, my stress decreased. Like all of these things, when you literally, when you take housing out of the equation, you can focus on so much more. And I was safe, and we were happy, and we were healthy. … In addition to a host of other things, I just never thought self-confidence, the budgeting, the financial aspect like I’ve received [several] promotions at my job since. So, it’s just made a huge difference, like when you’re stuck in a bad neighborhood, you really don’t see a way out….”

“I’ve been able to like basically almost double my income. So that’s…my major accomplishment.”

Lastly, participant reflection data suggested that stress levels appeared to decrease during the program period. Before participating, many described living with considerable stress due to precarity. By the end of the program, participants reported reduced stress, which they often associated with the combination of rental subsidies, neighborhood contexts, workshops, and coaching relationships. These observations informed the inclusion of stress reduction as an important component of the program logic model.

### Temporal Perspective of the Pathways to Family Economic Stability

The pilot program evaluation identified indications of financial improvement among participant families, alongside reports of enhanced housing stability, and mental health. Analysis of longitudinal data revealed a temporal pattern in the pathways through which families appeared to move toward economic stability (Figure 3). Reductions in stress appeared to serve as an important foundation for other positive changes.

**Figure 3.**
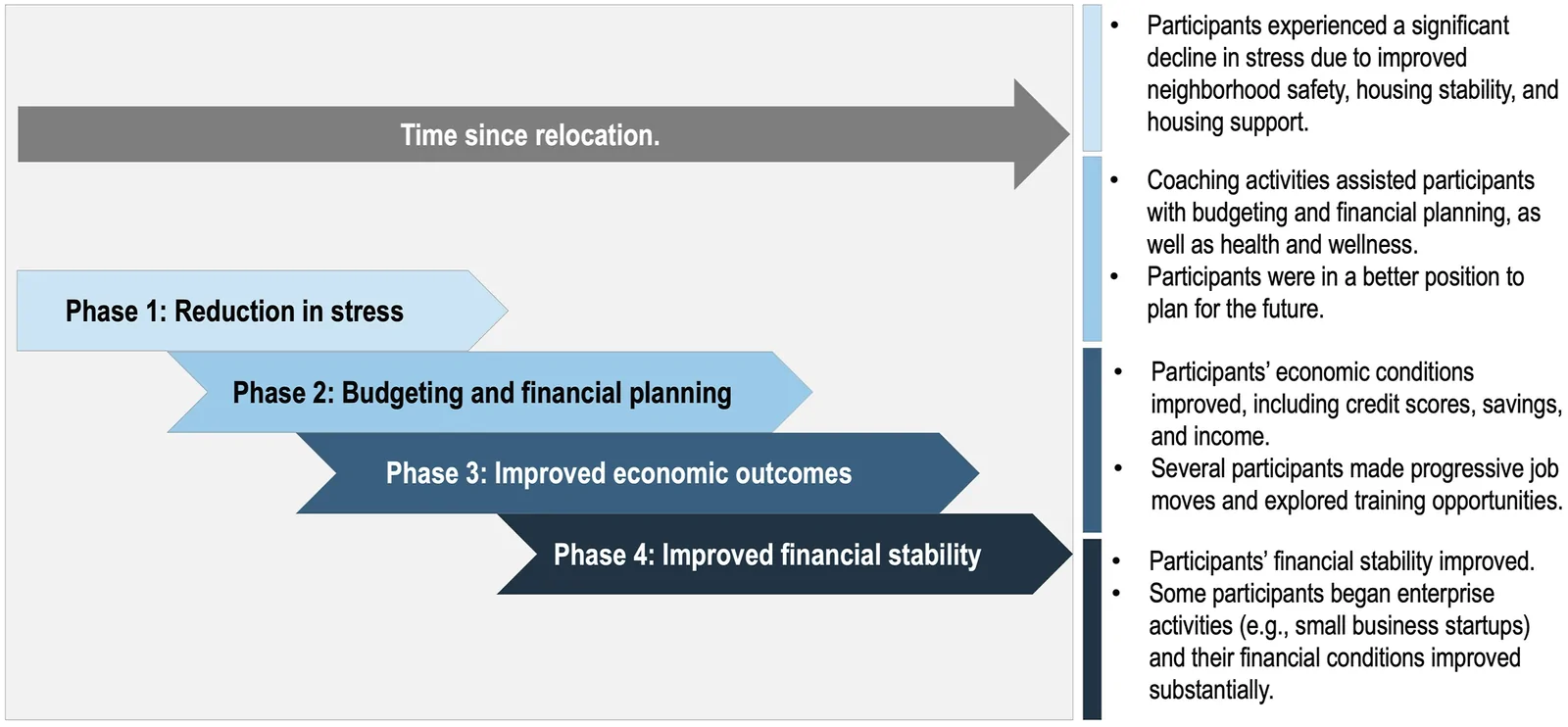
Temporal perspective on the pathways to achieving family economic stability. *Note.* Adapted from Reece et al. (2021, p. 23). The illustrated temporal sequence represents patterns observed in participants’ reports and should not be interpreted as an experimentally established causal order

In Phase 1, analysis indicated that relocation to a safe and more resourced neighborhood, coupled with improved housing conditions and housing security, appeared to correspond with an increased sense of safety and reduced stress among participants. These positive changes were accompanied by indications of improved children’s health, including fewer asthma attacks and enhanced emotional health.

Building on the stabilization observed in Phase 1, in the next two phases (Phases 2 and 3), participants appeared to have achieved greater stability and readiness to plan for the future, as reductions in stress may have enabled them to focus more on personal growth. Through interactions with coaches, new neighbors, and other program participants, they appeared to enhance their social capital. Participants commonly referenced various forms of social capital as a benefit of the coaching experience. Most reported that their economic circumstances had improved compared with their situation prior to entering the program, including better credit scores, increased savings, and higher income. Many also obtained certifications, earning promotions, or secured improved employment opportunities. Participants frequently identified the benefits of additional resources and wellness practices introduced through the monthly program. Taken together, these observations suggested that, along with life coaching, access to additional resources through the monthly programs and program partnerships may have supported participants’ development of financial and wellness skills.

In the final phase, participants appeared to demonstrate greater financial stability. Some engaged in entrepreneurial activities that corresponded with notable improvements in their financial conditions. According to administrative data, the majority of participants experienced gains in economic conditions, with an average annual income increase of 58% over the four-year pilot period.

As previous research shows that housing security and quality are associated with physical and mental health (Krieger & Higgins, 2002; Martin et al., 2019; Sandel et al., 2004; Singh et al., 2019; Stahre et al., 2015), this study observed positive changes in children’s health and parents’ mental health following improvements in housing security. Families who achieved stable housing appeared better able to pursue personal growth and reported improved employment conditions, in contrast to previous research showing that individuals experiencing housing insecurity due to forced displacement often face employment insecurity (e.g., Desmond & Gershenson, 2016).

### Program Logic Model

The logic model captures discussions from the program design and developmental phase, incorporating relevant insights gained from the formative evaluation and the interpretations of the temporal perspective on increasing family economic stability. At the center of the model is stress and its reduction. Families who live in precarity experience toxic stress, a stress experience which is chronic, severe, unmediated and unrelenting (Brisson et al., 2020). Toxic stress can cause significant health impacts at the family level and impact cognitive functioning. Figure 4 illustrates how the initial developmental model (Table 1) evolved into the final structure of the program logic model. The first and final sections in Table 1, titled *Households and Subsidy* and *Development and Research*, contributed to the overall program design and operation. In contrast, the second and third sections, *Social Supports* and *Housing*, transitioned into formal program components as depicted in Figure 4.

**Figure 4.**
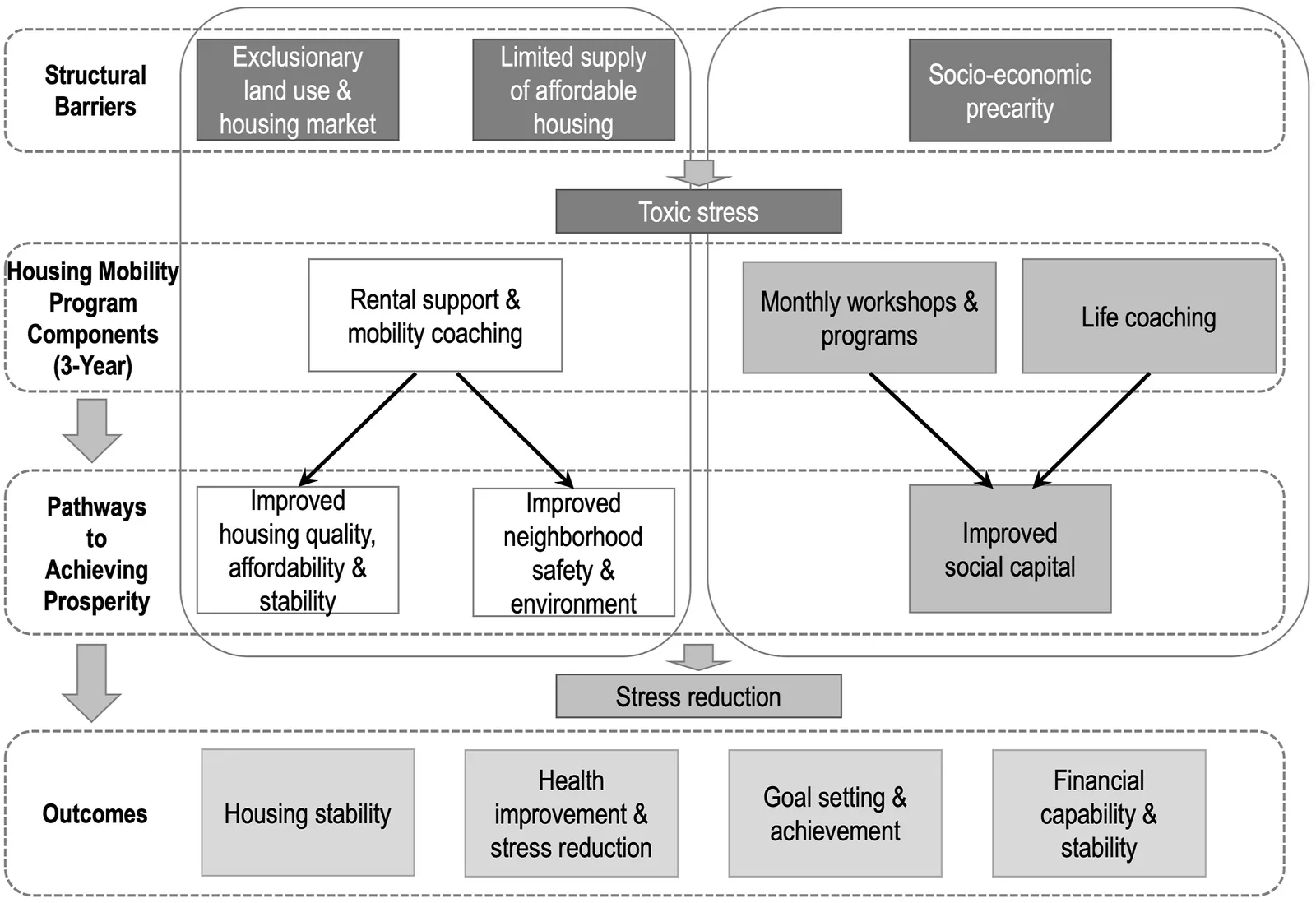
Program logic model

Building on the unexpected finding about the centrality of toxic stress, the program logic model identifies three structural barriers that contribute to toxic stress, three program components, and three pathways to achieving prosperity and outcomes.

The developmental evaluation identified three key structural barriers hindering the residential and financial stability of the target population in the program: exclusionary land use policies, a limited supply of affordable housing, and socio-economic precarity. First, exclusionary land use policies create significant obstacles for low-income families and communities of color, limiting their access to housing in high-opportunity neighborhoods. Affordable housing alternatives are mostly concentrated in low-opportunity communities, especially for Black households (Le et al., 2022). Second, low-income households are disproportionately affected by the challenges of housing affordability and the scarcity of housing subsidies. Over the past decade, the availability of affordable rental units has decreased, with a decline of 3.9 million units having contract rents lower than $600 (Joint Center for Housing Studies of Harvard University, 2023). Third, barriers related to accessing necessary information and resources through social capital further exacerbate these issues. For instance, not all eligible households with vouchers, who are permitted to move to high-opportunity neighborhoods, actually proceed with the move. This mobility decision can be explained by a lack of information, insufficient social capital, unpreparedness (Turner & Briggs, 2008), fear of discrimination, and stress (see Kleit et al., 2016). As a result, these low-income families may experience frequent moves, evictions, and housing instability (Kleit et al., 2016; Phinney, 2013).

To address these barriers, the pilot program implemented a multi-faceted intervention consisting of three core components over a three-year period: (1) a privately funded monthly rental support with mobility coaching, (2) monthly programs/workshops, and (3) life coaching. The monthly programs and life coaching focus on four key pillars aligned with each desired outcome: housing stability, improved health outcomes, education/career development, and financial capability and stability.

The three program components are designed to create pathways to achieving prosperity. Rental support and mobility coaching are intended to provide access to high-quality rental units in high-opportunity neighborhoods, which the program expects will improve housing quality, affordability, stability, as well as neighborhood safety and environment. Both monthly programs/workshops and life coaching are expected to strengthen participants’ social capital.

These three pathways are anticipated to reduce stress and ultimately contribute to four expected outcomes: housing stability, improved health, goal setting and achievement (e.g., education and career), and enhanced financial capability and stability.

## Discussion and Conclusion

This paper presents the development and progress of a new model of housing mobility program’s implementation and its immediate outcomes. We employed a developmental evaluation to create new program. We also used formative evaluation to aid in continued program improvement during the pilot program’s implementation.

Throughout both developmental and formative evaluations, we maintained an iterative approach. We developed a new program tailored to our target population and the short-term nature of this initiative, incorporating evidence-based best practices from programs focused on improving housing mobility (Darrah & DeLuca, 2014), supporting self-sufficiency (Brennan, 2014; Kleit, 2004; Rohe & Kleit, 1997, 1999), and providing financial coaching (NeighborWorks America, 2014). To develop the program, we assessed local context and politics, as well as the population of focus and their needs, by engaging stakeholders and partners, initiating a literature review, developing a program logic model, and designing the program. This developmental evaluation process required collaboration with a wide variety of stakeholders, partners, and the project team to develop the new program.

During the formative evaluation process, challenges faced during the pilot program’s implementation were addressed in weekly meetings. Prompt feedback helped to adjust and resolve issues, such as coach transitions, the impact of the pandemic, and bullying issues faced by children after relocation. Importantly, annual evaluation reports provided feedback on the program’s effectiveness and identified areas for improvement, whether short-term or long-term.

Based on promising findings from the pilot, this program was implemented as a demonstration program that would eventually support 100 families. The pilot ended in July 2022, and the demonstration program launched in September 2022 with 15 families. Two groups, each consisting of 16 to 18 families, have been joining each year. The pilot evaluation provided ongoing feedback and timely evaluations, which helped in updating the design of the demonstration program.

### Community-Engaged Approach to Program Design and Implementation

In addition to being informed by existing research literature, the Program design involved extensive community engagement with potential participants, service providers (e.g., landlords, coaches), various committees, and university partners. Community insights played a key role in shaping the initiative, based on the focus groups with potential participants, stakeholder roundtables, and ongoing feedback from multiple committees. These collaborative activities informed key program decisions, including the identification of high-opportunity neighborhoods and the development of coaching and monthly programs components. Through this community-engaged process, the program logic model reflected the needs and priorities articulated by community stakeholders. The initial developmental model was conceived in autumn 2016 and modified annually based on participatory input from program participants and other stakeholders.

Building on these activities, the Steering Committee—comprising service providers, local leaders, and university partners—guided several key governance decisions before and during the pilot. The committee decided that the pilot could commence when 70% of the three-year budget had been secured or pledged, ensuring financial viability. After communicating with program participants, the committee extended the pilot by one year in response to the COVID-19 pandemic’s impact on participants and their families and supported the shift from in-person to virtual meetings.

Unlike other housing mobility programs, such as MTO or Creating Moves to Opportunity, this Program added life coaching and monthly program components based on insights from focus groups to help participants navigate the unique challenges of a limited-term program. In a limited-term program, participants are keenly aware that their rental assistance will end and use the three years to plan accordingly. Therefore, with respect to post-move services, while the Community Choice Demonstration provides a one-month check-in and annual follow-ups, our Program offers ongoing life coaching and monthly programs/workshops over the three-year program period.

### Lessons Learned From the Evaluation

Throughout the process of developmental and formative evaluation, we learned that evaluator’s skill is a critical component. In developmental evaluation, evaluators are expected to not only adapt to emergent situations but also engage stakeholders in various ways (see Patton, 2011). This requires a combination of research and methodological skills (e.g., research design, evaluation theory, quantitative and qualitative methods, data analysis), as well as expertise in the area the program team or innovator aims to develop. Communication skills and expertise in engaging a variety of people from many walks of life were essential. In formative evaluation, evaluators also need methodological expertise and the ability to help the program team understand the evaluation findings and foster a learning environment (Patton, 2011).

When considering the replicability of these evaluation approaches, our experience suggests that developmental evaluation is more challenging to replicate than formative evaluation. This is because developmental evaluation is heavily dependent on the evaluator’s unique skills and capabilities. Previous literature highlights that certain characteristics of developmental evaluators can influence the effectiveness and utilization of evaluation results. These key attributes include “contribution to team, insightfulness, ability to communicate evaluation perspective, flexibility, analytical leadership” (Patton, 2008, p. 210), as well as “agile, open, interactive, flexible, observant, and have a high tolerance for ambiguity” (Patton, 2011, p. 336). Additionally, cultural competence, and the ability to build and sustain relationships with various stakeholders (e.g., program staff and participants) throughout many years are critical for maximizing the impact of developmental evaluations.

Additionally, evaluators should invest time and resources in developing and implementing a program through consistent meetings and communication over several years. Both evaluation approaches, particularly developmental evaluation, require considerable time and resources. For example, the program in this study took approximately two years from the initial discussions to the development of the program design and the start of implementation. During this period, the program team and university partners met weekly to discuss the local context, identify the target population and their needs, engage stakeholders and partners, and develop the program design. When partnerships exist between community and university, the university partner often contributes with little or no material benefits, viewing this work as a service to the community.

Additionally, when a university partner works with community partners, both partners need to find mutual benefit in all stages of evaluation design. Frequently the university partner is interested in publishing research, yet university partner might not be able to apply methodologically rigorous approaches that allow for attribution of causation of program outcomes. For example, the ideal evaluation design for this pilot program would have been a randomized experiment with a control group with a larger sample size. However, financing limits dictated a small sample size and community concerns about the impact of randomization on the participants made such a design impossible. In particular, community partners voiced the concern that these families were in crisis much of the time, and randomly assigning them to housing would heighten that trauma. Additionally, at the start of the program it was unclear how many families would meet entrance criteria, whether there would be an overabundance of families, and whether the program would find enough housing units to allow for random assignment. Thus, the evaluation became useful as a program development tool but less useful at attributing program impacts.

### Study Limitations

This study has two limitations when evaluating program outcomes, as detailed in the previous section. First, the sample size was constrained by feasibility and logistical issues. Second, due to similar capacity limitations, this evaluation did not include a control or comparison group, which somewhat limits the ability to assess impact. The evaluation findings were therefore interpreted cautiously, and a future randomized design has been proposed to more rigorously assess program impacts, as described in the ‘Implications for Housing Policy and Future Directions’ section.

Despite these limitations, the outcomes from this pilot program indicated positive patterns in participants’ financial circumstances and health following the provision of rental support in high-opportunity neighborhoods and access to comprehensive coaching. Moreover, annual evaluations were conducted using mixed methods and a longitudinal approach (i.e., baseline, three annual, and one exit surveys, and interviews), which captured experiences and measured outcomes for families to better understand the process.

### Implications for Housing Policy and Future Directions

Our work on developmental and formative evaluations provides a model for policy design that includes identifying the populations served by vouchers, defining the target population, and amplifying their voices. This is accomplished through initiating community dialogues, actively engaging with relevant topics, and maintaining structured discussions throughout the process to ensure inclusivity and responsiveness to the needs of the population.

Our approach has the potential to lead to a paradigm shift in policies and their implications, providing a more effective and holistic solution that addresses housing, health, and economic stability. A cost comparison between the two programs indicates that the annual cost of HCV appears lower than our program. This difference reflects the broader scope of services included in our program, such as coaching, monthly workshops, program design, and emergency support, in addition to rental and administrative costs. Our program supports are intended to promote greater financial stability within a time-limited housing support model. When considering the full duration of assistance, however, our program is less costly. HCV assistance continues indefinitely for eligible households, whereas our program provides housing support for three years.

Additionally, findings from this study suggest that participation in the Program, including its coaching and monthly meeting components, is associated with improvements in mental health and financial stability. These gains may translate into a reduced need for prolonged housing assistance and point to the potential for time-limited housing assistance paired with service supports to achieve long-term cost efficiencies relative to the HCV program. Policymakers might consider adopting limited-term housing support and life coaching instead of the current long-term housing subsidy model, given that federal rental assistance currently reaches only one in four renter households that qualify (Gartland, 2022). This shift could potentially reduce costs, enabling support for a larger demographic within the same budget, thus amplifying the impact on both housing security, health improvement, and economic stability for these families. Alternatively, the role of housing as a foundation in this work suggests that making voucher availability an entitlement could have long-lasting effects on poverty.

Based on participants’ experiences, the evaluation identified patterns suggesting that improvements in physical and mental health coincided with greater housing security and appeared to precede gains in employment, education, and income. These patterns suggest that improvements in family well-being and economic stability may be linked to the housing security and neighborhood safety experienced by participant families.

This study raises the following questions: Is the purpose of housing assistance limited to providing shelter, or are we aiming to achieve more extensive goals? While housing can relieve stress, addressing poverty requires more than providing shelter. To alleviate poverty, policymakers and practitioners should incorporate interventions beyond housing, including creating opportunities by improving neighborhood environments and expanding social capital.

For future research, this study could be expanded to strengthen causal inference regarding the Program’s impact on life outcomes through a quasi-experimental design involving a larger cohort of families and a comparison group. In such a design, all participants would meet the Program’s eligibility criteria, and program staff could assign eligible families to either the participant or comparison group. This approach would allow for the assessment of pre-, interim-, and post-program outcomes for both groups using annual surveys, interviews, and administrative data collected at regular intervals throughout the three-year program period and one-year post-completion. Additionally, future studies may benefit from exploring long-term outcomes for program families at regular intervals after the program’s completion.

## Supplemental Material

Supplemental Material - Insights From Developmental and Formative Evaluations: Developing and Implementing a Limited-Term Housing Mobility Program Supplemental Material for Insights From Developmental and Formative Evaluations: Developing and Implementing a Limited-Term Housing Mobility Program by Jee Young Lee, Jason Reece, Rachel Garshick Kleit in Evaluation Review.

Supplemental Material - Insights From Developmental and Formative Evaluations: Developing and Implementing a Limited-Term Housing Mobility Program Supplemental Material for Insights From Developmental and Formative Evaluations: Developing and Implementing a Limited-Term Housing Mobility Program by Jee Young Lee, Jason Reece, Rachel Garshick Kleit in Evaluation Review.

Supplemental Material - Insights From Developmental and Formative Evaluations: Developing and Implementing a Limited-Term Housing Mobility Program Supplemental Material for Insights From Developmental and Formative Evaluations: Developing and Implementing a Limited-Term Housing Mobility Program by Jee Young Lee, Jason Reece, Rachel Garshick Kleit in Evaluation Review.

Supplemental Material - Insights From Developmental and Formative Evaluations: Developing and Implementing a Limited-Term Housing Mobility Program Supplemental Material for Insights From Developmental and Formative Evaluations: Developing and Implementing a Limited-Term Housing Mobility Program by Jee Young Lee, Jason Reece, Rachel Garshick Kleit in Evaluation Review.

## Data Availability

The datasets generated and/or analyzed during the current study are not publicly available due to the presence of personal identifiable information but are available from the corresponding author on reasonable request.*

## Notes

1. Families Flourish Program (formerly Move to PROSPER or MTP) is referred to as “Program” throughout the manuscript.

2. In subsequent implementation of the program, both the state and local government have become supporters of the program, both politically and financially.
