# Supplementary material for: Insights From Developmental and Formative Evaluations: Developing and Implementing a Limited-Term Housing Mobility Program
Source: Eval Rev. 2026 Jul 16;50(6):1140–90. doi: 10.1177/0193841X261468394 (PMC13672924; doi:10.1177/0193841X261468394)
Supplement: Supplementary file 1 [file sj-pdf-1-erx-10.1177_0193841X261468394.pdf]

Thematic Analysis: Participant Interviews Fall 2018

Question 1: What inspired you to join the Program?

Desire to get child into a new neighborhood or school: child bullied, child unsafe, school quality poor, school not responsive to child's needs, distance from work, stressful environment, old neighborhood declining (X6)

Better living situation (housing) (X4)

Financial instability (X4)

Lower income but not low income enough for benefits (X3)

Recommended by another person (friend/boss/relative) (X3)

Looking for a (life) change (X3)

Attracted to the coaching opportunity (X1)

Question 2: How would you describe your experience in the program so far?

Positive experience, with some identifying the change as transformational. (X8)

Mixed experience. Positive but with some initial challenges. (X1)

Question 3A: Has the coaching been helpful?

Coaching has been very positive with benefits including: enhanced skills, greater socialization, relationship building with other participants, accountability. (X8)

Socialization

Relationship Building

Skill Development

Accountability

Coaching has been mixed experience, positive but some challenges: redundancy in skills I already have, frustration with participants showing up late to group sessions. (X1)

Question 3B: Are there particular goals you have worked on or achieved with your coach??

Budgeting/Financial Goals (X5)

Education and Scholarship Assistance (X4)

Parenting/Education Advocacy (X2)

Time Management (X1)

Question 4: How do you like your new neighborhood?

Very Positive: Cleaner, safer, more opportunity (X8)

Positive but some challenges: proximity (X1)

Question 5: How have your kids adjusted (into new schools and neighborhood)?

Very positive experience: children doing better academically, emotionally, are more engaged (X7)

Mixed experience: positive for some children, but at least one child having difficulty acclimating to new school (X2)

Parents who were initially apprehensive about the new school (X2)

Question 6: How is your experience with property management?

Positive Experience: Referencing friendliness, respectfulness, responsiveness and community events/interactions (X9)

- *Friendliness & Respectfulness: (X7)*
- Responsiveness: (X5)
- *Community Events and Positive Community Interactions: (X3)*

Question 7: How has your housing situation changed?

Improved: Poor Housing Conditions Prior to Move (X8)

- *Improved Maintenance and Cleanliness (X7)*
- *Improved Indoor Air Quality (Previous problems with mold and rodent infestation) (X4)*
- *More Living Space (X2)*

No Significant Change: Was Previously Living with Relative (X1)

Question 8: How as your financial situation changed?

Financial Situation Improved (X5)

- *Improved: Housing subsidy very important to stability and savings (X3)*

Improved: Avoided sacrificing job due to benefit cliff (X1)

Improved: Gift cards very helpful (X1)

No Change to Financial Situation (but financially stable) (X4)

Question 9: Have you experienced changes in you or your family's physical or mental health?

Improved Physical Health: (X4)

Improved Mental Health: (X8)

No Changes: (X1)

Question 10: What is the most important part of the program, the rental assistance, the move to a new neighborhood, the coaching, or all factors?

Multiple Program Elements: (8)

Primarily Coaching: (X1)

Question 11: What suggestions do you have to improve the program?

Improved Meeting Logistics: Timing, location; participation (X6)

Centralized Location & More Apartments (X1)

Opportunities to Volunteer in the Future (X1)

More Programming on Homeownership Opportunities (X1)

No Suggestions (X3)
