# Supplementary material for: Insights From Developmental and Formative Evaluations: Developing and Implementing a Limited-Term Housing Mobility Program
Source: Eval Rev. 2026 Jul 16;50(6):1140–90. doi: 10.1177/0193841X261468394 (PMC13672924; doi:10.1177/0193841X261468394)
Supplement: Supplementary file 2 [file sj-pdf-2-erx-10.1177_0193841X261468394.pdf]

Interview Questions for Participants:

1. What inspired you to join the program?
2. How would you describe your experience in the program so far?
3. Has the coaching been helpful?
    a. Are there particular goals you have worked on or achieved with your coach?
4. How do you like your new neighborhood?
    a. How does it compare to your old neighborhood?
    b. What has been your experience with your new neighbors and property management?
    c. Do your children like the new neighborhood?
5. Do you feel like your financial and housing conditions have changed since joining the program?
6. How have your children adjusted or responded to their new schools?
7. Have you noticed any other changes (positive or negative) with your children since relocating?
8. Has your health (physical or mental) changed since relocation?
9. What aspects of the program have been the most beneficial (in supporting your family)?
10. What aspects of the program have been most challenging (in supporting your family)?
11. What adjustments to the program do you think need to be made to support the families participating?
