# Supplementary material for: Insights From Developmental and Formative Evaluations: Developing and Implementing a Limited-Term Housing Mobility Program
Source: Eval Rev. 2026 Jul 16;50(6):1140–90. doi: 10.1177/0193841X261468394 (PMC13672924; doi:10.1177/0193841X261468394)
Supplement: Supplementary file 3 [file sj-pdf-3-erx-10.1177_0193841X261468394.pdf]

Interview Questions for Staff (Coaches), Leadership (Management) and Property Management:

1. Can you describe your role with the program?
2. How would you describe your experience so far?
3. How have the families responded to the program so far?
    a. Have you noticed any changes from the time you began until now?
    b. What do you think contributes to these changes?
4. How have families adjusted or responded to their new neighborhood?
5. How have families adjusted or responded to their new schools?
6. How have the families responded to the coaching process?
    a. What are some of the benefits and challenges of the coaching model?
7. Move to PROSPER has a unique program design, what have been the opportunities and challenges with using the program's unique model?
8. What are the most important aspects of the program design (in achieving program goals)?
9. What aspects of the program design have been challenging (in supporting program goals)?
10. What adjustments to the program design need to be made to support participants?
11. If you were able to plan the future for the Move to PROSPER program, what would you focus on? If the program evolves in the future, what should that look like?
