# Supplementary material for: Insights From Developmental and Formative Evaluations: Developing and Implementing a Limited-Term Housing Mobility Program
Source: Eval Rev. 2026 Jul 16;50(6):1140–90. doi: 10.1177/0193841X261468394 (PMC13672924; doi:10.1177/0193841X261468394)
Supplement: Supplementary file 4 [file sj-pdf-4-erx-10.1177_0193841X261468394.pdf]

Demographic Information:

1. What is your age? ______
2. What is your race/ethnicity?
    - White or Caucasian
    - Black/African American
    - Hispanic, Latino, or Spanish origin (please specify country of heritage/nativity in the space provided) ____________________
    - Asian (please specify country of heritage/nativity in the space provided)____________________
    - Native American/Alaska Native
    - Other/Multiple Races (please specify in the space provided) ____________________
    - Decline to answer
3. What was your income in the past year? _____________
4. What is the highest degree you have earned or level of school you have completed?
    - No schooling completed
    - Less than high school (please specify the highest grade that you completed in the space provided)
    - High school diploma
    - GED
    - Some college
    - 2-year college degree
    - 4-year college degree or beyond
5. What was your zip code prior to enrolling in Move to PROSPER? ___________
6. What is your current zip code? ____________

Move to PROSPER Experience

7. What inspired you to apply to Move to PROSPER? ______________________________
8. How would you describe your experience with the Move to PROSPER enrollment and admission process?
    - Very Negative
    - Negative
    - Neither Positive or Negative
    - Positive
    - Very Positive
    - Other Comments: ____
9. How would you describe your experience relocating?
    - Very Negative
    - Negative
    - Neither Positive or Negative
    - Positive
    - Very Positive
    - Other Comments: ____

10. How would you describe your experience with the Move to PROSPER coaches?
    - Very Negative
    - Negative
    - Neither Positive or Negative
    - Positive
    - Very Positive
    - Other Comments: ____
11. Has Move to PROSPER helped you achieve new goals?
    - Yes
    - No
    - Not Sure
    - If Yes, what goals were achieved: ____
12. How would you describe your experience in your new apartment?
    - Very Negative
    - Negative
    - Neither Positive or Negative
    - Positive
    - Very Positive
    - Other Comments: ____
13. What do you like most about your new apartment? _______________________________
14. What do you dislike about your new apartment? ________________________________
15. How would you describe your experience with property management?
    - Very Negative
    - Negative
    - Neither Positive or Negative
    - Positive
    - Very Positive
    - Other Comments: ____

Neighborhoods

16. Is this new neighborhood better, worse, or about the same as your last neighborhood?
    - Much worse
    - Worse
    - About the Same
    - Better
    - Much Better
    - Other Comments: ____
17. In your old neighborhood, how satisfied were you with…

	Very satisfied	Somewhat satisfied	Neither satisfied nor dissatisfied	Somewhat dissatisfied	Very dissatisfied
Neighborhood safety	5	4	3	2	1
Neighborhood cleanliness	5	4	3	2	1
Police protection	5	4	3	2	1
Friendliness of neighbors	5	4	3	2	1
Convenience to shopping	5	4	3	2	1
Access to public transportation	5	4	3	2	1
Access to your job or employment services	5	4	3	2	1
Convenience to church or other place of worship	5	4	3	2	1
Quality of your home	5	4	3	2	1
Amount of space in your home	5	4	3	2	1
How near you live to family and friends.	5	4	3	2	1

18. In your new neighborhood, how satisfied are you with…

	Very satisfied	Somewhat satisfied	Neither satisfied nor dissatisfied	Somewhat dissatisfied	Very dissatisfied
Neighborhood safety	5	4	3	2	1
Neighborhood cleanliness	5	4	3	2	1
Police protection	5	4	3	2	1
Friendliness of neighbors	5	4	3	2	1
Convenience to shopping	5	4	3	2	1
Access to public transportation	5	4	3	2	1
Access to your job or employment services	5	4	3	2	1
Convenience to church or other place of worship	5	4	3	2	1
Quality of your home	5	4	3	2	1
Amount of space in your home	5	4	3	2	1
How near you live to family and friends.	5	4	3	2	1

19. How much do you know now about the neighborhood you moved too?
    - A Little
    - Some
    - A Lot

- Other comments:____

20. At the time that you moved, how much did you know about what community services were available in the community you were moving to? (By community services, we mean things like day care centers, counseling agencies, health clinics, job training centers or welfare offices).
    - Nothing
    - A Little
    - Some
    - A Lot
    - Other comments:____
21. Do you want to know more about the community you moved too?
    - Yes
    - No
    - If Yes, what information would you like to know? _____________________________
22. Since moving to your new neighborhood have you had difficulty reaching any of the following destinations?
    - Work
        - Yes
        - No
        - Not a destination I travel too
    - School
        - Yes
        - No
        - Not a destination I travel too
    - Church or another place of worship
        - Yes
        - No
        - Not a destination I travel too
    - Shopping destinations
        - Yes
        - No
        - Not a destination I travel too
    - Family or friend's homes
        - Yes
        - No
        - Not a destination I travel too
23. For destinations you have difficulty reaching, what is the difficulty? (check all that apply)
    - It's too far away
    - You don't have access to transportation
    - Transportation is unreliable
    - Transportation is too expensive
    - Some other difficulty, describe: ____________________________
24. How would you describe your interactions with neighbors, other tenants, or property management staff such as complaints?

- Very negative
- Negative
- Neither negative or positive
- Positive
- Very Positive
- Other Comments: ____

25. Before moving to your current home, did you belong to any groups or associations, either locally or nationally such as charities, labor unions, professional associations, schools, political or social groups, sports or youth groups, and so forth?
    - Yes
    - No
    - Other Comments: ____
26. Since moving to your current home, have you belonged to any groups or associations, either locally or nationally such as charities, labor unions, professional associations, schools, political or social groups, sports or youth groups, and so forth?
    - Yes
    - No
    - Other Comments: ____

Economic and Housing Conditions

27. How would you describe your family's economic circumstances since relocating?
    - Much worse
    - Worse
    - Neither worse or better
    - Better
    - Much Better
    - Other Comments:____
28. How would you describe changes in your employment since relocating?
    - Much worse
    - Worse
    - Neither worse or better
    - Better
    - Much Better
    - Other Comments:____
29. Have you changed employment since relocating?
    - Yes
    - No
    - If Yes, why did you change employment? ___________________________
30. Have you had difficulty paying rent, utilities or other bills, since moving?
    - Yes
    - No
31. If you have had difficulty paying rent, utilities or other bills, since moving, how often (how many months) do you have these difficulties?

- Less than 2 months a year
- 2 to 4 months a year
- 4 to 8 months a year
- More than 9 months a year
- What bills do you have the most difficulty paying? ___________________________

32. How would you describe your family's housing circumstances since relocating?
    - Much worse
    - Worse
    - Neither worse or better
    - Better
    - Much Better
    - Other Comments:____
33. Has the quality of your housing improved has improved since relocating?
    - Yes
    - No
    - If yes, how is your new home better than your previous home?_____________________

Child Development and Family Well Being

34. How has your child adjusted (or reacted) to their new school?
    - Very poor adjustment/reaction
    - Poor adjustment/reaction
    - Neither poor or good adjustment/reaction
    - Good adjustment/reaction
    - Very good adjustment/reaction
    - Comments:____
35. How has your child's (or children's) behavior changed since enrolling in Move to PROSPER?
    - Very negative change
    - Somewhat negative change
    - No change
    - Positive change
    - Very positive change
36. How has your child's (or children's) grades changed since enrolling in Move to PROSPER?
    - Very negative change
    - Somewhat negative change
    - No change
    - Positive change
    - Very positive change
37. How has your child's (or children's) self image/self worth changed since enrolling in Move to PROSPER?
    - Very negative change
    - Somewhat negative change
    - No change
    - Positive change

- Very positive change

38. How has your child's (or children's) optimism changed since enrolling in Move to PROSPER?
    - Very negative change
    - Somewhat negative change
    - No change
    - Positive change
    - Very positive change
39. How has your child's (or children's) overall health changed since enrolling in Move to PROSPER?
    - Very negative change
    - Somewhat negative change
    - No change
    - Positive change
    - Very positive change
40. How many school absences has your child had since relocating (in the previous year)? ____
41. Overall how helpful has the program been to your children?
    - Very unhelpful
    - Unhelpful
    - Neither helpful or unhelpful
    - Helpful
    - Very helpful
    - Other comments:____

Health and Wellness

42. In general, would you say your physical health is:
    - Excellent
    - Very good
    - Good
    - Fair
    - Poor
43. Thinking about your physical health, which includes physical illness and injury, for how many days during the PAST 30 DAYS was your PHYSICAL health not good? ___
44. In general, would you say your mental health is:
    - Excellent
    - Very good
    - Good
    - Fair
    - Poor
45. Thinking about your mental health, for how many days during the PAST 30 DAYS was your MENTAL health not good? ___
46. During the PAST 30 DAYS, for about how many days did poor physical or mental health keep you from doing your usual activities, such as self-care, work or recreation? ____
47. How often do you get the social and emotional support you need?
    - Very rarely

- Rarely
- Sometimes
- Often
- Very often

48. Stress means a situation in which a person feels tense, restless, nervous or anxious or is unable to sleep at night because his/her mind is troubled all the time. Do you feel this kind of stress these days?
    - Not at all
    - Only a little
    - To some extent
    - Rather much
    - Very much
49. Has your physical health changed since relocating?
    - Very negative change
    - Somewhat negative change
    - No change
    - Positive change
    - Very positive change
    - Comments: ____
50. Has your mental health changed since relocating?
    - Very negative change
    - Somewhat negative change
    - No change
    - Positive change
    - Very positive change
    - Comments: _______________________________________
51. How has your stress changed since relocating?
    - Very negative change
    - Somewhat negative change
    - No change
    - Positive change
    - Very positive change
    - Comments: _______________________________________
52. About how long has it been since you last visited a doctor for a routine checkup?
    - Less than 6 months
    - 6 months to 1 year
    - 1 to 3 years
    - More than 3 years
53. In the past year, how many times have you or a member of your household been to the emergency room for medical care? ____ If more than 0, what was the cause or causes for ER visit:_____________
